# *Vitis vinifera* L. Leaf Extract, a Microbiota Green Ally against Infectious and Inflammatory Skin and Scalp Diseases: An In-Depth Update

**DOI:** 10.3390/antibiotics13080697

**Published:** 2024-07-26

**Authors:** Marta Armari, Elisa Zavattaro, Cesar Francisco Trejo, Alice Galeazzi, Alessia Grossetti, Federica Veronese, Paola Savoia, Barbara Azzimonti

**Affiliations:** 1 Laboratory of Applied Microbiology, Center for Translational Research on Allergic and Autoimmune Diseases (CAAD), Department of Health Sciences (DiSS), School of Medicine, Università del Piemonte Orientale (UPO), Corso Trieste 15/A, 28100 Novara, Italy; marta.armari@uniupo.it (M.A.); 20033123@studenti.uniupo.it (A.G.); 20028046@studenti.uniupo.it (A.G.); 2 Dermatology Unit, Department of Health Sciences (DiSS), School of Medicine, Università del Piemonte Orientale (UPO), Via Solaroli 17, 28100 Novara, Italy; elisa.zavattaro@med.uniupo.it (E.Z.); federica.veronese@med.uniupo.it (F.V.); paola.savoia@med.uniupo.it (P.S.); 3 Roelmi HPC Srl, Via Celeste Milani 24/26, 21040 Origgio, Italy; cesar.trejo@roelmihpc.com

**Keywords:** anti-inflammatory activity, antimicrobial activity, dysbiosis, fermentation, opportunistic pathogens, postbiotics, skin and scalp diseases, skin microbiota, *Vitis vinifera* L. leaf extract

## Abstract

The skin microbiota, with its millions of bacteria, fungi, and viruses, plays a key role in balancing the health of the skin and scalp. Its continuous exposure to potentially harmful stressors can lead to abnormalities such as local dysbiosis, altered barrier function, pathobiont overabundance, and infections often sustained by multidrug-resistant bacteria. These factors contribute to skin impairment, deregulation of immune response, and chronic inflammation, with local and systemic consequences. In this scenario, according to the needs of the bio-circular-green economy model, novel harmless strategies, both for regulating the diverse epidermal infectious and inflammatory processes and for preserving or restoring the host skin eubiosis and barrier selectivity, are requested. *Vitis vinifera* L. leaves and their derived extracts are rich in plant secondary metabolites, such as polyphenols, with antioxidant, anti-inflammatory, antimicrobial, and immunomodulatory properties that can be further exploited through microbe-driven fermentation processes. On this premise, this literature review aims to provide an informative summary of the most updated evidence on their interactions with skin commensals and pathogens and on their ability to manage inflammatory conditions and restore microbial biodiversity. The emerging research showcases the potential novel beneficial ingredients for addressing various skincare concerns and advancing the cosmeceutics field as well.

## 1. Introduction

**Figure 1:**
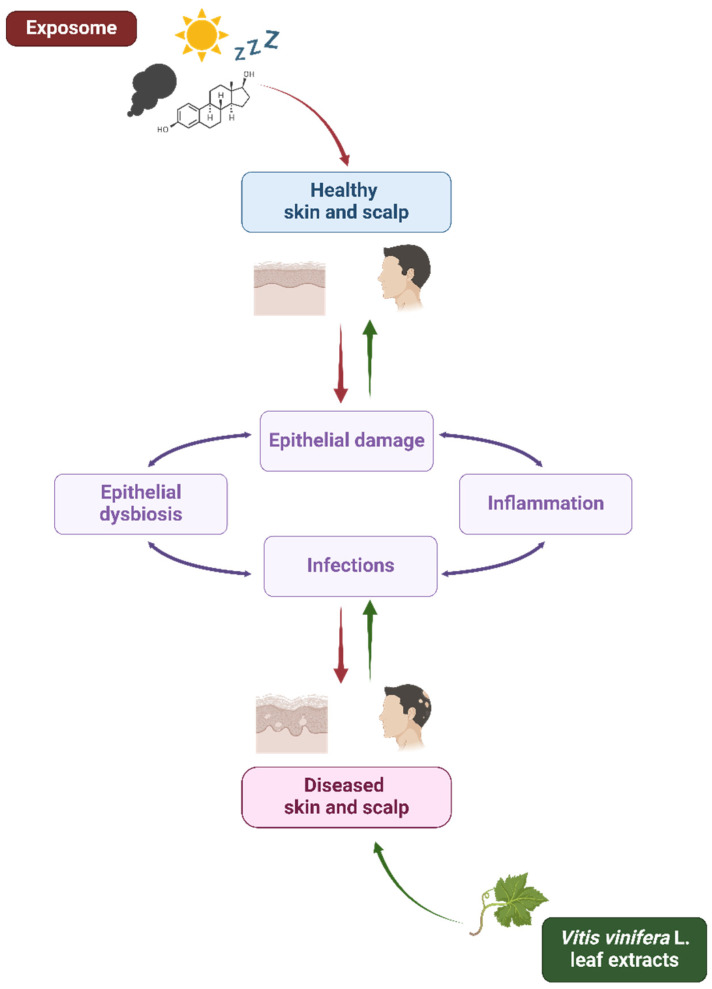
Exposome and *Vitis vinifera* L. leaf extracts’ influence on skin and scalp health. Schematic representation of the interplay among the exposome, *Vitis vinifera* L. leaf extracts, and epithelial barrier in skin and scalp health. The exposome affects the epithelial integrity contributing to microbial dysbiosis, inflammation, and infections (red arrows). On the other hand, *V. vinifera* L. leaf extracts could contribute to microbial eubiosis and skin and scalp health (green arrows). Created with BioRender.com.

The skin, the outermost selective barrier of the human beings, exerts its protective role through physical, chemical, biochemical, and immunological mechanisms [1]. Exposure to harmful external stressors (such as pollutants, ultraviolet (UV) radiation, and tobacco), alongside the influence of physiological internal factors (like aging, hormones, and sleep) and the host genetic background, can be responsible for the disruption and function impairment of this tissue, as already deeply investigated by many authors (Table 1) [2,3]. According to the extended “epithelial barrier hypothesis” introduced by Cezmi Akdis, epithelial damage may be associated with the onset and worsening of local microbial dysbiosis and infectious and inflammatory conditions [4] interconnected by bidirectional dynamic interactions [5,6,7]. As a matter of fact, a weakened barrier means opportunistic pathogen overcolonization, virulence expression, and infectious events, thus contributing to microbiota alteration, host immune response dysregulation, and chronic inflammation, leading to a vicious cycle of aggravating pathological events (Figure 1) [8,9].

Gut epithelial disruption and dysbiosis have both local and systemic consequences, not only gastrointestinal [5,10] but also neurodegenerative, cutaneous, psychiatric, metabolic, and many others [4,11]. Likewise, epidermal impairment and microbiota alterations are linked to skin and scalp disorders like acne, atopic dermatitis (AD), psoriasis, vitiligo, hidradenitis suppurativa (HS), seborrheic dermatitis (SD), and alopecia areata (AA) [12,13,14,15,16,17,18] but also to systemic conditions such as systemic lupus erythematosus (SLE) [19], thanks to the gut–skin and gut–brain–skin axis relationship [20,21].

To manage these conditions, the preferred topical therapies may include anti-inflammatory drugs, such as corticosteroids [14,22]. Although they are not free from side effects, the risk is minimal when properly used [23]. However, ideal topical remedies should not interfere with the host skin eubiosis to avoid any possible pathological self-reinforcing vicious circuit [24,25]. In this scenario, novel effective strategies are needed.

Due to the presence of plant secondary metabolites (PSMs), plants and derived extracts have been effectively used for the treatment of several human health conditions [26]. Among the PSMs, polyphenols are the most promising ones because of their recognized antioxidant, anti-inflammatory, antimicrobial, anticancer, and immunomodulatory properties [27,28,29].

*Vitis vinifera* (VV) L. or grapevine is an emblematic example of polyphenols and other PSM-rich plants [30,31]. Agro-industrial biomasses such as grapevine leaves produced by the wine industry are an interesting raw material for the pharmaceutic, cosmeceutics, and nutraceutical markets, since they contain bioactive compounds that can be extracted and exploited in accordance with the bio-circular-green economy model needs [32,33,34]. Till now, only scarce research evidence has been available on both their anti-inflammatory activity, especially in reconstructed skin models, and on their interaction with the skin microbiota [35,36,37,38].

**Table 1:** Overview of the main factors involved in skin impairment and disorders.

General Domains	Specific Factors	References
External stressors	Solar radiations	[39,40,41]
	Environmental factors (e.g., pollutants, allergens, particulate matter, ozone, industrial toxic gases, nano/microplastics, pesticides, tobacco …)	[8,40,42,43,44,45]
	Climate change	[8,41,42,43,44,45]
	Nutrition	[3,40,46,47]
Internal factors	Hormones (e.g., sex hormones, thyroid hormones, glucocorticoids, …)	[40,48,49]
	Psychological stress	[50,51,52,53]
	Sleep	[40,54]
	Aging	[55,56]
	Host microbiota/microbiome and microbial exposome	[57,58]
Host genetic background (eukaryotic cells)	Polymorphisms and other mutations	[59,60,61]
	Epigenetics	[60,61,62]
	Ethnicity	[41,63]
	Biological sex	[64,65,66]

On theses bases, the aim of this review article is to provide an informative updated summary on the potential of VV, specifically of its leaf component, starting from its derivatives and active ingredients, including resveratrol and pterostilbene, in the prevention and management of inflammatory conditions affecting skin and scalp, and regarding its interactions with the skin microbiota as well as its activity against opportunistic infections. Future perspectives and limitations of this approach are reported as well.

## 2. Materials and Methods

In this literature review, the MEDLINE NIH National Library of Medicine (NLM)’s electronic free medical bibliographic PubMed and Scopus publicly available databases were consulted by looking for the keywords and terms “*Vitis vinifera* L. leaf extract”; “microbiota”; “microbiome”; “skin microbiota”; “skin microbiome”; “skin virota”; “skin bacteriota”; “skin mycobiota”; “gut-skin axis”; “bacteria”; “virus”; “epitheliotropic virus”; “mycetes”; “fungi”; “dermatophytes”; “*Staphylococcus* spp.”; “Human Papillomavirus”; “Human Herpes virus”; “*Candida* spp”; “*Malassezia* spp.”; “inflammation”; “inflammatory skin diseases”; “inflammatory scalp diseases”; “seborrheic dermatitis”; “ psoriasis”; “atopic dermatitis”; “hidradenitis suppurativa”; “acne”; “immune system”; “cytokines”; “cosmeceutics”; and “dermocosmetics”, both in single and/or in combination, and the Medical Subject Heading (MeSH) controlled vocabulary, in paper titles and abstracts to retrieve the highest quality indexed and most cited original peer reviewed scientific research studies and reviews.

The bibliographic literature search was focused on original scientific international studies written in the English language and recently published, with the older ones not exceeding the last 25 years (final consultation in mid July 2024).

## 3. *Vitis vinifera* L. Leaves

VV plays a prominent role in global agriculture as one of the most significant perennial crops. It has been acknowledged worldwide for its versatility, providing not only grapes for wine and alcoholic beverages but also a variety of other products, including raisins, juices, fermented foods, cosmeceutics, and nutraceuticals, among others [31,67].

Nowadays, it is imperative to harness all by-products of the VV production chain to achieve a sustainable approach from ethical, economic, and environmental perspectives. The reuse of these biomasses also contributes to waste reduction and promotes more ethical and environmentally friendly practices [68,69]. Among these winery discarded materials, grapevine leaves, similarly to other by-products such as pomace, are rich in PSMs, namely polyphenols, as well as lipids and organic acids [70]. Due to the health-promoting properties of said compounds, e.g., in terms of antioxidant and anti-inflammatory potential, their exploitation could guarantee many advantages, such as the production of novel active ingredients through biotechnological processes, while contributing to the green circular economy model [71,72]. Even though PSMs can potentially be found in every plant organ, some plant by-products are more likely to be investigated than others. Indeed, grape leaves have been studied less with respect to other raw materials, mainly in terms of extraction and utilization of PSMs [70,73].

### 3.1. Phytochemical Profile and Bioactive Compounds

As previously introduced, *Vitis vinifera* L. leaf extracts (VVLEs) are rich sources of diverse bioactive compounds, mainly PSMs. These phytochemicals have health-promoting properties and are integral to the plant adaptive strategies, serving as defenses against infections and key contributors to the grapevine’s ability to thrive in its environment [74,75,76]. The extracts are particularly abundant in polyphenols, encompassing a wide spectrum of compounds such as anthocyanins, catechins (including catechin, epicatechin, gallocatechin, and epicatechin3-O-gallate), ellagitannins (like brevilagin-1, vitilagin, and isovitilagin), and flavones (traces of quercitrin, quercetin, kaempferol, rutin, isoquercitrin, and luteolin). The presence of various organic acids, such as tartaric, malic, oxalic, fumaric, succinic, citric, and glyceric, contributes to the overall chemical complexity. Phenol acids, such as the o- and p-hydroxybenzoic, protocatechuic, gallic, vanillic, syringic, and ellagic, further enhance the bioactive profile. Esters, featuring cinnamic and tartaric acids, add another layer of chemical diversity. The extracts also contain essential vitamins, including C, PP, B group, and folic acid. Carotenoids, volatile constituents, proteins, and mineral salts (ranging from 5% to 7%) overall offer a versatile array of bioactive compounds with potential applications in various health-related contexts [77,78].

To sum up, the main classes of VVLE compounds include phenolic acids, flavonols, flavonoids, stilbenes, lignans, tannins, and anthocyanins (Table 2 and Table 3). However, in terms of qualitative and quantitative characterization, their phytochemical profile is strongly influenced by several factors. Environmental stimuli are involved, such as soil properties, weather conditions, and the season during which leaves are harvested. Grape cultivars and varieties, stage of maturation, and plant health status are responsible for the production of different PSMs, too [37,79,80,81,82,83]. Interestingly, although the phenolic compounds’ characterization in grapevine berries has been extensively studied, only limited information is available regarding the polyphenols profile in the leaves and their biological activities.

### 3.2. Topical and Systemic Applications in Cosmeceutics

According to the World Health Organization (WHO), herbal medicines encompass herbs, herbal materials, preparations, and finished herbal products containing parts of plants or other plant materials, or combinations thereof. Their use is well established and widely recognized as safe and effective for the treatment of various health conditions [109]. The ancient tradition of herbal medicine has given rise to phytomedicines, appreciated for their low cost and reduced toxicity compared to other synthetic products [110,111].

In this context, VV leaves (VVLs) have been employed due to their diverse biological activities (e.g., anti-inflammatory, immunomodulatory, anticancer, and anti-infectious) and are widely recognized as natural antioxidants and chemopreventive agents [112]. The polyphenol content and the related biological activities make VVLEs interesting for many applications not only in the pharmaceutical market but also in the cosmetics one [113,114,115]. VVLEs in cosmetics products mainly serve as anticaries, antidandruff, antioxidant, and antipathogen agents [77,116]. In more detail, VVLEs help combat free radicals [117] while contributing to the maintenance of the natural microbiota of the skin and scalp [118]. Additionally, they protect against the harmful effects of UV radiation, positioning them as potential ingredients in sunscreen filters [119]. Although many products are currently available in the cosmetics and personal care markets, the underlying mechanisms of action and health-promoting properties of these raw materials and derived ingredients are still under investigation.

## 4. Skin Microbiota and Inflammatory Skin and Scalp Disorders

### 4.1. Skin Microbiota and Healthy Skin

The skin is globally recognized as a selective barrier. This function is guaranteed by the dynamic crosstalk between its microbiota and the below epidermal, dermal, and hypodermal strata, mainly through the secretion of mediators like antimicrobial peptides (AMPs), cytokines, and chemokines [57,120]. Being an interface, the skin coordinates the responses to external and internal both xenobiotic (e.g., toxic substances, UV rays, and opportunistic pathogens) and familiar stimuli (Figure 1) to maintain or restore skin microbial and immune balance, as well as to reduce the onset and progression of inflammatory local and systemic conditions. In this way, it is possible to avoid or solve skin disorders and ensure tissue homeostasis, appearance, and hydration [40]. To do this, the mechanisms underlying these processes should be investigated. A key role is played by the local microflora, recognized as one of the five functional levels of the skin as reviewed by Lefèvre-Utile et al. [121]. Since the launch of the Human Microbiome Project (HMP) in 2007, the microbial communities that colonize the skin are under investigation and the research on this topic has exponentially grown over the years [122]. Commensal and pathogenic microorganisms have been identified, while their relationships with the host and the changes during life are less known [123,124]. Human skin commensals (i.e., bacteria, fungi, and viruses) are in mutualistic relationships with their host, since they are involved in the development and regulation of the immune system, while hindering endogenous and exogenous infections [125,126]. However, in dysbiotic conditions, this fine orchestra no longer works [127,128].

### 4.2. Skin Microbiota and Cutaneous Diseases

The first evidence suggesting the role of microbiota in skin diseases dates to more than 20 years ago, with the so-called “hygiene hypothesis” stating that a low exposure to pathogens during childhood correlates with a high incidence of allergic disorders [129]. Subsequently, some attempts in preventing or lowering the incidence of atopic dermatitis (AD) and food allergies have been developed with oral probiotics’ administration in pregnant women and newborns, with no univocal results [130]. Through the years, this hypothesis has been further extended with the introduction of the “old friends hypothesis” by Graham Rook and the “biodiversity hypothesis” by Tari Haahtela. Nowadays, mechanisms described by these three theories are included in the more comprehensive “epithelial barrier hypothesis” by Cezmi Akdis, which could help understand the etiopathogenesis of several dermatological diseases, like AD and many others [4,131,132].

AD, a chronic and relapsing skin disorder characterized by intense itching and eczematous lesions, is a part of the so called “atopic march”, that involves different organs and develops sequentially with age [133]. Although its pathogenesis is multifactorial, skin dysbiosis is crucial, with a relative abundance of *Staphylococcus aureus* and local inflammation. Furthermore, the raised presence of the yeast *Malassezia* spp. contributes to its severity [134].

These species are also a causative agent of folliculitis in sebaceous gland-rich sites and are involved in seborrheic dermatitis (SD), a common and relapsing disease located in sebaceous body areas (i.e., scalp, central area of the face, chest, and upper part of the back), manifesting as yellowish and greasy scales on erythematous skin. Despite that its pathogenesis is not fully elucidated, cutaneous dysbiosis seem to be involved. Specifically, *Malassezia* spp. hydrolyze the sebum triglycerides into lipid peroxides and free fatty acids, thus favoring inflammation and the yeast growth itself. Moreover, *S. aureus* has also been found over-represented in SD subjects, thus underlying the role of microbiota changes in both such dermatitises [135,136].

A further chronic inflammatory dermatosis is acne, in which *Cutibacterium acnes* (previously known as *Propionibacterium acnes*) plays a key pathogenic role, since it promotes keratinocyte differentiation, autophagy, and response to different stimuli, thus leading to a cytokine-mediated pro-inflammatory pattern and further immune cell recruitment. Furthermore, specific *C. acnes* strains can induce biofilm formation and promote bacterial adhesion, thus reducing any antibiotic effect [137]. *C. acnes* is affected by the presence of the commensal *Staphylococcus epidermidis*, that, reducing inflammation, is thus linked to less severe forms of acne [138].

Several anaerobic microorganisms, such as *Corynebacterium*, *Prevotella*, *Porphyromonas*, and *Peptoniphilus* spp., are commonly detected in hidradenitis suppurativa (HS), a chronic and relapsing disease of the apocrine glands manifesting with abscesses leading to scarring and fistulae mainly in the axilla, groin, and gluteal regions. Such bacteria produce damage-associated molecular patterns (DAMPs), allowing the inflammatory process amplification through cytokines and pattern recognition receptors (PRRs). On the contrary, a reduced count of *S. epidermidis*, *Staphylococcus hominis,* and *C. acnes* has been reported, while their replenishing helps the recovery [139,140].

Psoriasis is a chronic and relapsing inflammatory skin disease characterized by occurrent scaling and erythematous plaques [141]. Its pathogenesis is multifactorial and is linked firstly to dendritic cell activation, followed by the production of cytokines and promotion of Th1 and Th17 patterns, followed by keratinocyte proliferation and dermal inflammation. The bacterial strains on plaques are mainly *Corynebacterium*, *Propionibacterium*, *Staphylococcus,* and *Streptococcus* spp.; accordingly, a causal interaction between streptococci and eruptive guttate psoriasis had been suggested. On the other side, even a possible role of *Malassezia* spp. has been hypothesized, especially in relapses; thus, the yeast might upregulate the expression of specific cytokines. Furthermore, the sebo-psoriasis variant could represent a further element supporting the role of *Malassezia* spp. in this context [142].

Concluding, the mentioned diseases have distinct patterns, which involve some specific microorganisms that are summarized in Table 4.

## 5. Inflammatory Skin and Scalp Disorders and *Vitis vinifera* L. Leaf Extracts

The potential use of VV for skin and scalp diseases’ management, not only directly linked to microbial alterations, represents a current field of investigation in dermatology, despite that most of the studies are related to the grape seed extracts (GSEs). Recently, Soleymani et al. have reviewed the therapeutic effects of GSEs, and, mainly, of the nanoformulated resveratrol in some inflammatory skin disorders (i.e., acne, facial redness, chloasma, and chronic venous insufficiency), as well as in wound healing and skin aging [143]. Indeed, resveratrol possesses several health-promoting effects besides its anti-inflammatory potential, but its content in berries and leaves depends on the genetic background of the cultivars and on the plant organs as well [144,145].

To date, data on VVLEs are still scarce. Sangiovanni et al. recently reported their in vitro anti-inflammatory effect in cell cultures mimicking skin disorders. The authors found that in HaCaT cells, all the bioactive substances of water extract from VVL (VVWE), and not its singular main components, significantly reduced the IL-8 release induced by the NF-κB pathway following tumor necrosis factor-alpha (TNF-α), lipopolysaccharide (LPS), or ultraviolet B (UVB) stimulus, thus stating a synergism, while a lack of effect was detected in response to hydrogen peroxide (H_2_O_2_) [35].

As information regarding VVLEs is still poor, some authors investigated the effects of specific phytochemicals found in the grape leaves, as well as in other VV organs. Among them, in a recent review, Marko and Pawliczak properly evaluated the impact of resveratrol in psoriasis and AD. They reported the positive effects of local resveratrol in such skin diseases, but they also suggested that the in vitro results should be cautiously considered, given the difficulties in reproducing psoriasis and AD in animal models faithfully. Moreover, they did not consider any interaction between microbiota and resveratrol [146]. In addition, a clinical case on the effectiveness of oral isoquercetin (a flavonoid also contained in VV) in a man complaining of prurigo nodularis complicating AD has been reported by Pennesi et al. [147].

When considering the oral administration of grape derivatives, some authors reported their beneficial effects in different skin diseases, including AD in a mouse model and acne. In this latter disease, a randomized clinical trial involving patients treated with oral isotretinoin for severe acne showed that in subjects receiving both isotretinoin and a dietary supplement containing vitamins and VV, the acne clinical scores improved, and a greater adherence to the therapy with lower side effects was achieved [148,149]. Unfortunately, the precise composition of the administered supplementation was not specified.

Moreover, the beneficial effect of pterostilbene, found not only in VVLs but also in other plant sources such as blueberries, has been investigated in AD models. Interestingly, in an AD-induced mouse model, its topical application decreased skin thickness, evaluation scoring, and IgE levels [150].

When considering microbiota and acne, VVLE var. *aglianico* has been demonstrated as the most active plant derivative to inhibit the growth of *C. acnes*, but no effect was documented in countering the biofilm production [151]. Moreover, in HaCaT and THP-1 cells stimulated by *C. acnes*, quercetin suppressed pro-inflammatory cytokine production (i.e., IL-1β, IL-6, IL-8, and TNF-α) via TLR2 and MAPK pathways and decreased the MMP-9 expression. In addition, the anti-inflammatory effect of quercetin was tested in a mouse model intradermally injected with *C. acnes*, where it significantly reduced the cutaneous erythema and swelling [152].

Furthermore, VV derivatives demonstrated positive effects in numerous randomized clinical trials involving HS-affected patients, but the activity of the investigated product, represented by GSE administered orally, was only evaluated for the HS-associated metabolic syndrome improvement [153].

Only few reports exist on the use of VV derivatives in scalp disorders, namely in SD and hair loss. It has been reported that grape seed compounds of VV can counteract the *Malassezia* spp. activity in SD [154,155]. Accordingly, in hair loss, the potential use of GSE has been investigated both in vitro and in vivo. More in detail, proanthocyanidins and procyanidins from GSE were tested for local use both in hair cultures and in C3H mice, thus showing an increased hair proliferation both in cultures and after topical application in mice, probably due to the telogen-anagen phase conversion in the hair cell cycle [156]. The above-mentioned substances and their possible effects in dermatological disorders are summarized in Table 5.

From a molecular point of view, only few studies analyzed the effect of VVLEs in cutaneous inflammatory pathways, mainly focusing on their potential protective role towards UV damage [38]. Firstly, VVLE pre-treated HaCaT cells were separately irradiated with UVA and UVB. UVA increased DNA damage and cell cycle S phase block, thus leading to apoptosis even in the presence of VVLEs. UVB irradiation decreased cell viability and increased apoptosis not counteracted by VVLEs; this effect is probably due to the possible extract difficulties in acting as an antioxidant and the reduced pre-treatment time. However, these extracts demonstrated a protective activity against UV-induced damage [36]. Moreover, when evaluating keratin expression in UVB-induced skin damage (both in HaCaT and 3D normal skin organotypic cultures), keratin 17 (K17) expression induced by the pSTAT3 pathway significantly decreased in the presence of VVLEs. Since K17 can be considered as a reliable marker of UVB damage in skin, the authors concluded that VVLEs in vitro can protect from UVB-mediated epidermal injury [157]. Secondly, when pre-treated with VVLEs, UVA-irradiated normal human dermal fibroblasts showed an increased cell viability through the activation of SIRT1 and HSP47, two genes involved in the anti-aging processes, suggesting a further potential effect of the extracts in such condition [158]. Moreover, collagen XVII, a hemidesmosome transmembrane protein whose role has been recently studied both in skin and hair aging, as well as in wound repair and blistering skin diseases, increased in human skin models treated with pterostilbene and its glucoside [159,160]. Given the abundance of such chemicals in VVLEs, a further potential application in dermatology could be speculated.

Lastly, no data are currently available regarding the potential sensitization and irritant effect of VVLEs in skin, scalp, and mucosal surfaces (i.e., eyes and genitalia). More in general, a recent review reported that both VV fruit, juice, and seed extracts are non-irritant or only minimally irritant for the skin. Accordingly, fruit, seed, and grape skin extracts were classified as non-irritant or mildly irritant for the ocular surfaces [34].

## 6. *Vitis vinifera* L. Leaf Extracts, Microbiota, and Skin Opportunistic Pathogens

Among their properties, many authors highlight how polyphenols may inhibit or enhance microbial growth and survival, depending on the phytochemical structure and on the bacterial species [161]. Overall, they can exert a double beneficial effect, since they may counteract pathogen invasion and virulence, while supporting beneficial strains [162,163].

When used for human health, a key aspect to be stressed is that polyphenols establish a bidirectional interaction with the host microbiota, especially the gut one [37,164]. Due to their poor absorbability in the digestive system, once they arrive in the large intestine, they can act as prebiotics, thus modulating the human microbiota composition [162,165]. At the same time, polyphenols can be substrates of bacterial enzymes and undergo a process of biotransformation, being released in the bloodstream and thus conferring local and systemic beneficial effects also through the gut–brain and gut–skin axes [161,166,167,168].

In this context, several works focus on VV L. polyphenolic compounds and their activity. Different plant organs (i.e., fruits, leaves, seeds, skin, canes, stems, and roots) have distinct phytochemical profiles, as summarized by Sharafan et al. [34]. Consequently, extracts from different plant parts also have different antimicrobial effects: for instance, seed extracts have shown a greater antimicrobial potential compared to leaf ones [169]. In particular, a correlation between the total phenol content and the resulting antimicrobial activity has been highlighted, but several other factors (such as cultivar, environmental stimuli, and genotype) are also involved in determining the final effects [170,171,172].

**Figure 2:**
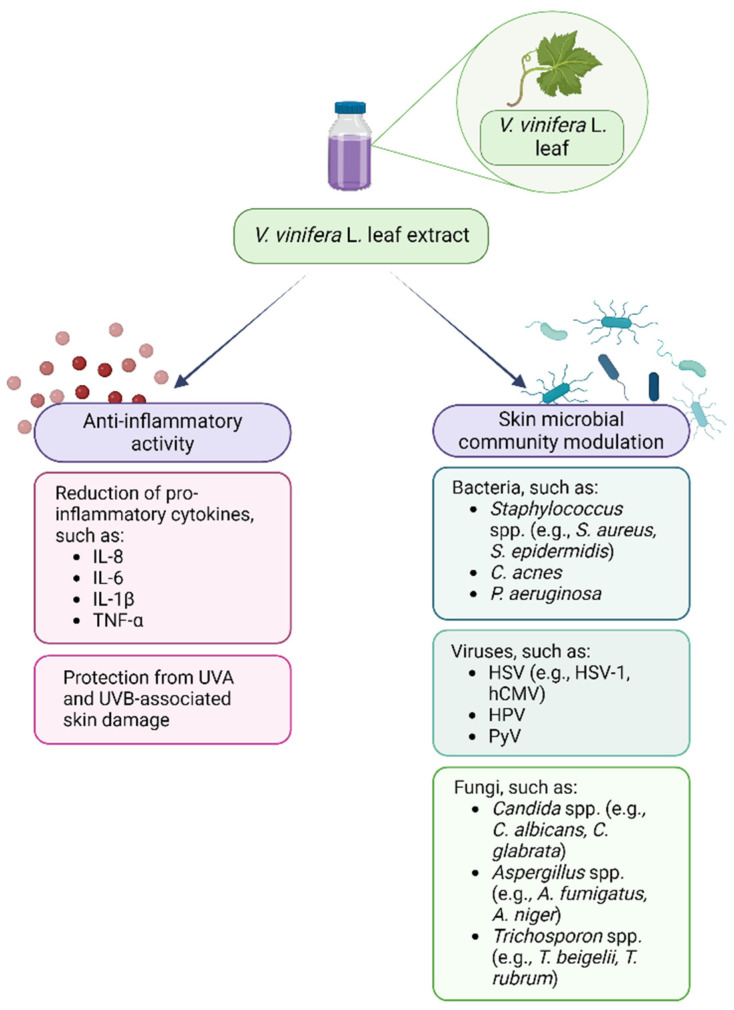
*Vitis vinifera* L. leaf extract anti-inflammatory and skin microbial community modulatory activity. Created with BioRender.com.

Therefore, plant polyphenols may be used in support to or as alternatives to the currently available antimicrobial drugs that nowadays have limited efficacy due to the global spread of antimicrobial resistance and to their ability to reduce the microbiota diversity [173,174]. Regarding the interaction between VVLEs and skin microorganisms, even if the antibacterial, antiviral, and antifungal properties of these extracts and polyphenols have been demonstrated in vitro on selected pathogenic microorganisms, the literature about the effects on the skin microbiota is very poor, so this topic needs to be further investigated. Lastly, the anti-inflammatory and prebiotic potential could synergistically enhance the beneficial effects of these compounds in the management of dysbiotic conditions (Figure 2). As a matter of fact, PSMs are known for their potential in restoring and improving gut dysbiosis by increasing the abundance of beneficial commensal that could counteract the inflammatory microenvironment [175,176]. This evidence, alongside the knowledge of their benefits on skin and hair health, could support the need for further investigation regarding their interaction with the skin microbiota [177].

### 6.1. Vitis vinifera L. Leaf Extracts and Bacteria

The antibacterial potential of VVLEs has been tested on different microorganisms, usually opportunistic pathogenic ones, some of which are also involved in skin infections. Gram-positive bacteria like *Staphylococcus* spp. seem to be more susceptible to the antibacterial activity of VVLEs if compared to Gram-negative bacteria, such as *Pseudomonas aeruginosa* [80,178,179]. The different sensitivity to phenolic-rich extracts has often been reported, regardless of the grape raw material [172,180,181]; nevertheless, other studies showed that there is not any statistically significant difference in response between Gram-positive and -negative bacteria [79,182,183]. Discording data may be attributable both to the extracts and the bacteria. On the one hand, as previously stressed, each extract has a distinct phytochemical profile which can result in different outcomes, and the solvents they are dissolved in may interfere with the biological response; on the other one, bacteria show species-specific and also strain-specific sensitivity to treatments [181,184,185]. The different susceptibility between Gram-positive and Gram-negative bacteria could also be linked to the bacterial surface and structure. Gram-negative double-layered cell envelope and periplasmic enzymes, e.g., some isoforms of metallo-β-lactamases (MBLs), protect the bacteria against many antimicrobial drugs by hindering their diffusion and degrading them, respectively [186,187].

Interesting results have been presented by Andelković et al. in 2015. Their study highlighted how extracts from grapevine leaves of two different varieties, Merlot and Vranac, had a significant antimicrobial activity towards Gram-positive bacteria more than Gram-negative ones. Notably, different activity depended not only on the grapevine variety, but also on the health status of the raw material itself. Extracts from *Plasmapora viticola*-infected leaves showed an increased antimicrobial effect when compared to those extracted from healthy leaves, probably due to the different PSM production of healthy and unhealthy plants [80].

In addition to the general antibacterial effects on planktonic forms, antibiofilm potential of VVLEs on skin pathogens is being investigated as well. Since currently available antimicrobial therapies are not always effective against biofilm forms, it is imperative to find new strategies for their prevention, treatment, and containment [188,189]. Grapevine leaves may have antibiofilm potential due to the presence of resveratrol and pterostilbene, of which antimicrobial and antibiofilm activity have been demonstrated on selected bacteria [190]. Stilbenoids, the phenolic group resveratrol and pterostilbene belong to, are classified as phytoalexins and as such are usually synthetized by the plant in response to infections or other damages [191]. These compounds seem to be more active against Gram-positive bacteria, but systematic studies are still needed to better understand their effects [192]. Lately, pterostilbene has gained more and more interest thanks to its pharmacological properties that make it a better candidate for therapeutic and clinical uses than resveratrol for the management not only of infectious disorders but also of dermatological issues [193,194].

Coenye et al. have identified the potential of resveratrol in counteracting the growth and biofilm formation of *C. acnes*, involved not only in the etiopathogenesis of acne vulgaris but also in implant-associated infections [195,196]. On the other hand, as previously reported in this review, Nelson et al. tested VV L. var. *aglianico* leaf extracts on *C. acnes* and highlighted their ability to inhibit bacterial growth without exerting any cytotoxicity on HaCaT cells, but they were not as effective in the eradication of mature biofilm forms. However, resveratrol was not detected in the selected extracts, despite the evidence in the literature [151].

Other studies were conducted on *S. epidermidis*, *S. aureus*, and *P. aeruginosa*, three of the main skin pathobionts involved in wound-associated biofilms, chronic wounds, and unpaired healing among other morbidities [197,198]. Even if antimicrobial effects are microorganism-dependent, both resveratrol and pterostilbene appeared to be more effective against Gram-positive bacteria, having shown low or no efficacy on *P. aeruginosa* [189,199]. Pterostilbene showed a greater potency than resveratrol not only in the treatment and prevention of biofilm but also in the inhibition of planktonic forms of Gram-positive cocci, namely, *S. epidermidis*, *S. aureus*, vancomycin-intermediate *S. aureus* (VISA), methicillin-resistant *S. aureus* (MRSA), and also *Enterococcus faecalis* [199,200]. Although none of these studies involved grapevine leaves or other winery by-products as sources for resveratrol and pterostilbene, it could be worthy to further delve into VVLEs and their phytochemical profile in order to understand the possible synergistic interactions among these and other secondary metabolites and the effects on the skin microbial communities.

### 6.2. Vitis vinifera L. Leaf Extracts and Viruses

Among VV-associated therapeutic properties exerted on microorganisms, both commensal and pathogenic ones, the antiviral effects have been investigated as well.

As reported in *Viruses*, Zannella et al., after examining the VVL content via an HPLC-MS/MS analysis, demonstrated the ability of the 40 identified phenolic compounds to inhibit the replication of the human opportunistic cutaneous Herpes simplex virus type 1 (HSV-1) pathogen (and also that of the severe acute respiratory syndrome coronavirus 2, SARS-CoV-2) in vitro in the very early infection phases of an eukaryotic Vero cell line model. Through plaque reduction assays and different experimental conditions (i.e., co-treatment, virus pre- and post-treatment, and cell pre-treatment), the authors showed that the leaf extracts were able to inhibit the virus–host cell binding and all the succeeding infection stages, as shown by the lack of gene expression involved in the viral infection cycle [201]. In another research study, aimed to assess the antimicrobial and antioxidant properties of VV aqueous leaf extract fractions, the authors confirmed that the phenolic compounds (i.e., terpenoids, flavonoids, and other related molecules) contained in the chloroform (CHCl_3_) fraction have potential antiviral effects towards the HSV-1 DNA virus (and the RNA Parainfluenza virus, PIV). Experiments were conducted once again in the Vero cell line model, in which viral strain titers were assessed with the Frey and Liess protocol as 50% tissue culture infectious dose (TCID50) using acyclovir and oseltamivir as per internal controls. These data are really promising, thus also supporting the extract being employed in folk medicine [178].

In the study of Berardi et al. on murine Polyomavirus (Py), chosen as the model system, resveratrol showed dose- and time-dependent in vitro antiviral activity expressed after the virus’s entry into the target cells. The authors suggested that this effect could be linked to its ability to inhibit the murine Py DNA synthesis in mouse normal fibroblast 3T6 cells, probably due to the damage caused to the plasma membrane. As a result, the virus translocation from the endoplasmic reticulum to the nucleus, and hence the viral DNA production, were both hindered. This phenomenon was observed also in the human tumor HL60 cell line. Therefore, the resveratrol presence seems to be needed till the end of the productive cycle to completely inhibit the viral DNA synthesis, translation, and eventually the progeny production [202].

Despite not being specifically demonstrated for grape leaves, resveratrol also abrogated the DNA replication of the human Cytomegalovirus (hCMV), responsible for reactivating skin infections in immunocompromised elderly and/or transplanted people, as demonstrated by its ability of inactivating in vitro the epidermal growth factor in human embryonal fibroblasts [203].

Regarding SARS-CoV-2, even though it does not directly infect the skin, it may lead to mucocutaneous visible signs in infected patients, mainly in the form of hyperpigmentation, a common inflammatory dermatological disorder characterized by extra melanin production after injury, irritation, or antibiotic use. Since melanin production is regulated by tyrosinase and many bioactive compounds of VV act as tyrosinase inhibitors (e.g., resveratrol, gallic acid, chlorogenic acid, epicatechin, and rutin), these PSMs could be exploited for their antimelanogenic potential [204,205].

Notably, the skin virota also includes beta and gamma Human Papillomavirus (HPV), which may promote non-melanoma skin carcinogenesis in transplant and/or immunosuppressed patients [206]. The inhibitory potential of resveratrol against mucosal high-risk (HR) HPV16 and HPV18 E6 and E7 oncoproteins, involved in cervical carcinogenesis, was demonstrated in vitro on HeLa and CaSki cells [207]. Data on cutaneous HPVs are not yet available, but this could be worthy of further investigation.

### 6.3. Vitis vinifera L. Leaf Extracts and Fungi

The antifungal activity of VVLEs has been explored but, worthy of note, only sporadic clinical reports can be retrieved from the scientific literature. Indeed, the potential effect of VVLEs on dermatophytes (i.e., fungi affecting skin and appendages, mainly causing superficial infection), molds, and yeasts has been investigated in various studies evaluating different single chemical compounds. In particular, the analyzed groups of substances belonging to the VVLEs were mainly represented by flavonols and stilbenes, both abundant in grapevine leaves [83]. As already reported for the antibacterial and antiviral activities, pterostilbene and resveratrol are the two compounds that have showed interesting antifungal properties in vitro. Resveratrol was found to act as fungicidal agent on *Candida albicans*, by interfering with the ergosterol biosynthesis, but it demonstrated a lack of effectiveness towards other *Candida* spp., such as *C. glabrata*, *C. parapsilosis*, *C. tropicalis*, *C. krusei,* and *C. dubliniensis* [208]. The high antifungal activity of pterostilbene has been demonstrated both in vitro and in vivo, being fivefold higher than resveratrol, and was suggested to be related to its better bioavailability and high capability to penetrate hydrophobic membranes, such as pathogen biofilm. In more detail, pterostilbene significantly inhibited biofilm formation in yeasts like Trichosporon cutaneum and, less weakly, *C. krusei*, while no effect was observed in *C. albicans* and *C. parapsilosis* [200]. In order to counteract this issue, pterostilbene was incorporated in poly-lactic-co-glycolic acid nanoparticles (PLGA NPs) and its effect on anti-*C. albicans* biofilm formation was compared with that of the free form and the crude pomace extract, demonstrating a significant superiority of PLGA NPS in allowing the delivery of antifungal drugs [209].

Resveratrol was found to be active in vitro against two other species of yeasts (*Saccharomyces cerevisiae* and *Trichosporon beigelii*), towards which its potency was comparable to Amphotericin B. Its mechanism of action was reported to induce apoptosis through the activation of metacaspases and cytochrome C release. Moreover, resveratrol was able to inhibit up to 80% of the growth of some of the most common human dermatophytes (i.e., *Trichophyton mentagrophytes*, Trichophyton rubrum, *Trichophyton tonsurans*, *Microsporum gypseum*, and *Epidermophyton floccosum*). When considering pathogenic molds, resveratrol was also tested against *Aspergillus niger* and *Aspergillus fumigatus*, responsible for various diseases among immunocompromised patients, demonstrating different degrees of activity depending on concentrations and VV cultivar [210].

Some studies have also reported the antifungal effect of other substances that can be found in the VVLEs, though these effects were observed through experiments involving different herbal plants. Indeed, epigallocathechin-3-gallate from green tea was found to inhibit the folate pathway and, also, ergosterol biosynthesis, both leading stopping the *C. albicans* growth, and a potential synergy with traditional treatments (azoles and terbinafine) was also suggested [211]. The same yeast was also targeted by caffeic acid, via inhibition of the isocitrate lyase enzyme and, consequently, lowering its virulence capability. Lastly, the dermatophyte *T. rubrum* development was demonstrated to be hindered by gallic acid and quercetin with a mechanism involving the activity of sterol 14-α-demethylase P450 and the expression of FAS1 and ERG6, respectively, thus interfering with the ergosterol synthesis [210].

## 7. *Vitis vinifera* L. Extract Prebiotic and Postbiotic Properties

The need for novel and/or alternative approaches for the management of skin inflammation in dysbiotic conditions has opened the doors to several new possibilities. As a matter of fact, due to the great health-promoting potential of grapevine and derived biomasses, their exploitation could provide novel active ingredients able to counteract pathogenic microorganisms and to support the local microbial flora. For instance, grapevine leaves could be converted into high-value-added products through biotechnological processes. In this context, the development of white biotechnology has emerged as a transformative force since it relies on enzymes and microorganisms to produce valuable components from renewable sources for the food, pharmaceutical, and cosmeceutics markets as well as other industries [212,213].

Recently, precision fermentation and the study of bioprocesses for the development of innovative active ingredients obtained through such procedures have been demonstrated to be a valid strategy to valorize many waste materials, satisfying the needs of the bio-circular-green economy model [214,215]. One application is represented by postbiotics, defined as “preparations of inanimate microorganisms and/or their components that confer a health benefit on the host” by the International Scientific Association for Probiotics and Prebiotics (ISAPP) in 2019 [216]. Postbiotics have unique properties that could be exploited in several sectors (e.g., food, dermocosmetics, and pharmaceutical markets), thus opening new scenarios and possibilities, such as the improvement in many skin issues, both pathological (i.e., AD) and aesthetic ones (i.e., wrinkles) [116,217].

Indeed, by understanding the basic scientific mechanisms of how these products work, it is possible to design more specific ones that address a wide range of functions, providing end users with reliable and effective solutions that could potentially prevent or improve pre-existing skin and scalp issues.

Due to their phenolic content, VVLEs could be exploited both for their prebiotic properties and for the development of postbiotics. Even if not completely understood yet, the interaction between polyphenols and microbiota is bidirectional, with the microbiota metabolizing polyphenols which themselves may modulate its quality, quantity, variety, and functions [37]. As a result, VVLEs are ideal prebiotic candidates for further processing in postbiotic production through probiotic-driven fermentation, as already reported in the literature for other raw materials [217,218]. Thanks to their greater specificity of action on the resident microbiota, and their interaction with the host cells, fermented extracts could better improve microbiome-based skin conditions compared to non-fermented ones [219]. Probiotic precise fermentation closely mimics the physiological processes of conversion of dietary polyphenols by the gut microbiota. In more detail, it leads to the ex novo or over-production of bioactive molecules, including phenolic-derived postbiotics and exopolysaccharides (EPSs), known for their health-promoting benefits, ranging from microbial restoration to immunomodulatory properties, anti-inflammatory, antiproliferative, and antioxidant ones [219,220,221]. Additionally, precision fermentation technology allows for more controlled bioprocesses and parameters, thus generating high-value-added products. Evidence suggests that fermented biomolecules, produced by the natural metabolism of microorganisms, also improve microbiome modulation [222,223]. However, despite productivity and yield being continuously optimized to ensure the quality and efficacy of the final products, this biotechnological approach still poses safety and standardization challenges, since many parameters can affect the final product features [224,225,226]. Lastly, their properties and phytochemical profile also depend on the selected fermenting microorganisms and on the natural substrate itself, here represented by VVLs [37,227].

## 8. Conclusions

The recent advances in dermatological and microbiota-related research highlight the importance of microbiota-friendly approaches to ensure eubiosis, while counteracting inflammatory conditions. For this reason, greater attention is devoted to plant-based bioactive compounds also because of their action dualism. If on one side they exert skin anti-inflammatory and antioxidant effects, on the other, they reduce pathogens, while sustaining beneficial microbial species at the same time. The exploitation of plant biomasses and the production of plant-derived ingredients through microbe-driven fermentation could represent a great opportunity for the management of many inflammatory dysbiosis-related skin and scalp issues (e.g., AD, SD, acnes, HS, and psoriasis).

Despite that *Vitis vinifera* L. and its leaves appear to be promising to help solve these conditions, the mechanisms of action, the effects on skin commensals and pathogens, and the synergistic forces underlying the observed health-promoting benefits are yet to be fully established. Moreover, their bioavailability, penetrating capability, and half-life, as well as the optimal administration route and standardization of extraction, isolation, and fermentation processes, also need to be investigated.

## Figures and Tables

**Table 2:** Phytochemical profile of *Vitis vinifera* L. leaves.

Phytochemical Class	Group	Main Compounds	References
Flavonoids	Anthocyanins	delphinidin-3-O-glucoside, cyanidin-3-O-glucoside, petunidin-3-O-glucoside, peonidin-3-O-glucoside, malvidin-3-O-glucoside, petunidin-3-(6-O-acetyl)glucoside, peonidin-3-(6-O-acetyl)glucoside, malvidin-3-(6-O-acetyl)glucoside, cyanidin-3-(6-O-coumaroyl)glucoside, petunidin-3-(6-O-coumaroyl)glucoside, peonidin-3-(6-O-coumaroyl)glucoside, malvidin-3-(6-O-coumaroyl)glucoside	[37,75,80,83,84,85,86]
	Flavan-3-ols	gallocatechin, catechin, procyanidin A1, procyanidin B1, procyanidin B2, procyanidin B3, procyanidin B4, epicatechin, epigallocatechin, epigallocatechin gallate, gallocatechin gallate, epicatechin gallate, catechin gallate	[37,75,80,83,85,87,88,89,90,91]
	Flavonols	quercetin, quercetin-3-O-glucoside, kaempferol, myricetin, myricetin-3-O-galactoside, myricetin-3-O-glucuronide, myricetin-3-O-glucoside, quercetin-3-O-rutinoside, quercetin-3-O-galactoside, quercetin-3-O-glucuronide, myricetin-3-O-rhamnoside, quercetin-3-O-rhamnoside, kaempferol-3-O-galactoside, kaempferol-3-O-rutinoside, kaempferol-3-O-glucuronide, quercetin-3-(6-O-acetyl)glucoside, quercetin-3-(3-O-arabinosyl)glucoside, quercetin-3-(7-O-glucosyl)glucuronide, kaempferol-3-O-glucoside, kaempferol-3-O-xyloside, kaempferol-3-O-rhamnoside, isorhamnetin-3-O-galactoside, isorhamnetin-3-O-glucoside, quercetin-3-(6-O-rhamnosyl)galactoside, isorhamnetin-3-O-arabinose, isorhamnetin-3-O-glucuronide, isorhamnetin-3-O-rutinoside, isorhamnetin-3-(4-O-rhamnosyl)rutinoside, kaempferol-3-(6-O-coumaroyl)glucoside, kaempferol-3(7-O-glucosyl)galactoside, diquercetin-3-(3-O-glucosyl)glucuronide, quercetin-3-O-galactoside, quercetin-3-O-glucuronide, quercetin-3-O-glycoside	[37,75,80,83,85,87,88,89,90,91]
Phenolic acids	Hydroxybenzoic acids	parahydroxybenzoic acid, protocatechuic acid, vanillic acid, gallic acid, syringic acid	[37,75,80,83,91,92,93]
	Hydroxycinnamic acids	caffeic acid, caftaric acid, caffeic acid, trans-caftaric acid, trans-coutaric acid	[37,75,80,83,91,92,93]
Stilbenes and their derivatives	Stilbenes derivatives, simples, glicosiled stilbenes, dimeric stilbenes	resveratrol, trans-piceid, trans-resveratrol, cis-resveratrol, trans-ε-viniferin, pterosilbene	[94,95,96,97,98]
Coumarins	Furanocumarins or simple cumarins	aesculin, fraxin, aesculutin, umbelliferone	[83,93]
Lignans	Monocyclic lignans Bicyclic lignans Neolignans Furofuran lignans cedrusin and its glucosides	isolariciresinol, lariciresinol, secoisolariciresinol	[99,100]

**Table 3:** Chemical structure and mechanism of action of *Vitis vinifera* L. leaves’ phytochemical compounds.

Phytochemical Group	Structure	Mechanism of Action	Reference
Anthocyanins	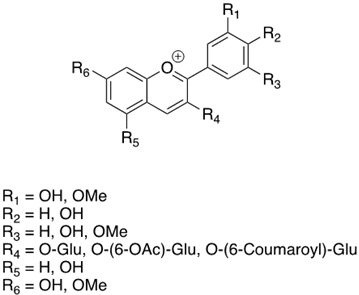	In vitro inhibitory activity towards growth and biofilm formation of *S. aureus* through quorum sensing disruption	[101]
Flavan-3-ols	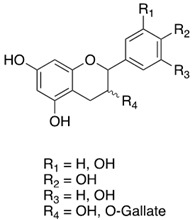	Antioxidant activity by free radical scavenging, transition metals chelation, as well as enzyme mediation and inhibition Antimicrobial and antiviral effects	[102]
Flavonols	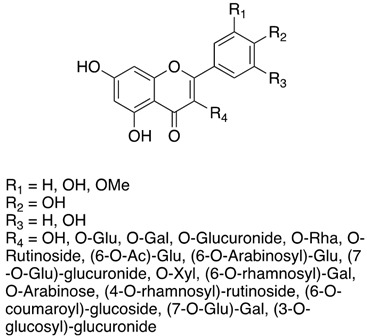	Protection from oxidative stress Radical species quenchers both via hydrogen atom (HAT) and electron transfer (ET) Phenolic OH groups may undergo deprotonation, thus reacting with free radicals at a faster rate according to a sequential proton loss electron transfer (SPLET) mechanism	[103]
Phenolic acids	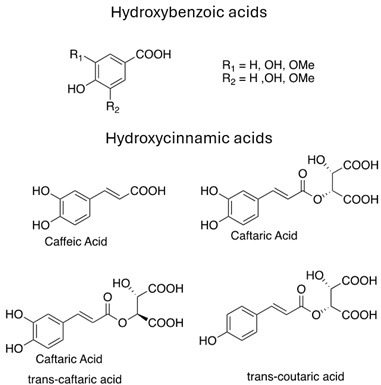	Antimicrobial property against multidrug resistant pathogens through hyper acidification on the plasma membrane	[104]
Stilbenes	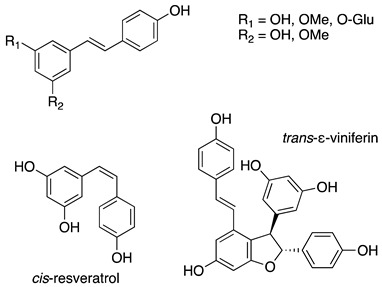	Broad pharmacological and biological activities: anticancer, antimicrobial, anti-aging, antioxidant and anti-inflammatory Anti-inflammatory activity through pro-inflammatory cytokines’ inhibition such as TNF-α and IL-1	[105]
Coumarins	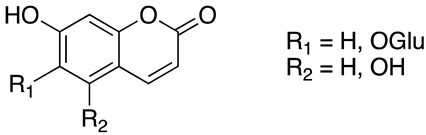	Photoprotective effect. UV absorbers, with photo-oxidative, antioxidant, and photosensitizing properties The conjugation reaction of coumarins with UV light induces bacteria death and virus inactivation	[106,107]
Lignans	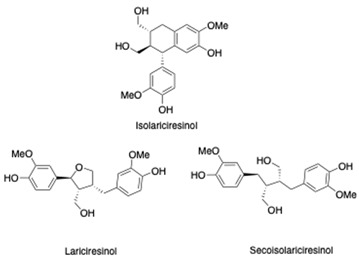	Anti-inflammatory and antioxidant properties Inhibition of ROS-induced activation of the NF-kB pathway.	[108]

**Table 4:** Skin and scalp disorders with microbial involvement.

Skin and Scalp Disorders	Main Microorganisms Involved	References
Atopic Dermatitis	↑ *S. aureus* ↑ *Malassezia* spp.	[133,134]
Seborrheic Dermatitis	↑ *Malassezia* spp. ↑ *S. Aureus*	[135,136]
Acne	↑ *C. acnes*	[137,138]
Hidradenitis Suppurativa	↑ *Corynebacterium* spp. ↑ *Prevotella* spp. ↑ *Porphyromonas* spp. ↓ *S. aureus* ↓ *S. epidermidis* ↓ *C. acnes*	[139,140]
Psoriasis	*Corynebacterium* spp. *Propionibacterium* spp. *Staphylococcus* spp. *Streptococcus* spp. ↑ *Malassezia* spp.	[142]

↑ more abundant microrganisms, ↓ less abundant microrganisms.

**Table 5:** Skin and scalp disorders and *Vitis vinifera* L.

Skin and Scalp Disorders	Use Evidence of VV or Its Bioactive Compounds	References
Atopic Dermatitis	Assessment of resveratrol activity on in vitro cell models Administration of resveratrol in animal models	[146]
	Oral administration of isoquercitin in a patient with prurigo nodularis complicating AD	[147]
	Topical application of pterostilbene on AD-induced mouse models	[150]
Seborrheic Dermatitis	Assessment of GSE activity on *Malassezia* spp.	[154,155]
Acne	Oral supplementation of vitamins and VV in patients receiving isotretinoid	[148]
	Assessment of quercitin on in vitro cell models Topical administration of quercitin in a mouse model	[152]
Hidradenitis Suppurativa	Oral administration of GSE for HS-associated metabolic syndrome in animal models and in randomized clinical trials	[153]
Psoriasis	Assessment of resveratrol activity on in vitro cell models Administration of resveratrol in animal models	[146]
Hair loss	Topical administration of proanthocyanidins and procyanidins from GSE in hair cell cultures and C3H mice	[156]

## References

[1] Baroni A., Buommino E., De Gregorio V., Ruocco E., Ruocco V., Wolf R. (2012). Structure and function of the epidermis related to barrier properties. Clin. Dermatol..

[2] Wild C.P. (2012). The exposome: From concept to utility. Int. J. Epidemiol..

[3] Celebi Sozener Z., Ozdel Ozturk B., Cerci P., Turk M., Gorgulu Akin B., Akdis M., Altiner S., Ozbey U., Ogulur I., Mitamura Y. (2022). Epithelial barrier hypothesis: Effect of the external exposome on the microbiome and epithelial barriers in allergic disease. Allergy.

[4] Akdis C.A. (2021). Does the epithelial barrier hypothesis explain the increase in allergy, autoimmunity and other chronic conditions?. Nat. Rev. Immunol..

[5] Weiss G.A., Hennet T. (2017). Mechanisms and consequences of intestinal dysbiosis. Cell. Mol. Life Sci..

[6] Hrncir T. (2022). Gut Microbiota Dysbiosis: Triggers, Consequences, Diagnostic and Therapeutic Options. Microorganisms.

[7] Balato A., Cacciapuoti S., Di Caprio R., Marasca C., Masarà A., Raimondo A., Fabbrocini G. (2019). Human Microbiome: Composition and Role in Inflammatory Skin Diseases. Arch. Immunol. Ther. Exp..

[8] Pat Y., Ogulur I., Yazici D., Mitamura Y., Cevhertas L., Küçükkase O.C., Mesisser S.S., Akdis M., Nadeau K., Akdis C.A. (2023). Effect of altered human exposome on the skin and mucosal epithelial barrier integrity. Tissue Barriers.

[9] Chopra D., Arens R.A., Amornpairoj W., Lowes M.A., Tomic-Canic M., Strbo N., Lev-Tov H., Pastar I. (2022). Innate immunity and microbial dysbiosis in hidradenitis suppurativa—Vicious cycle of chronic inflammation. Front. Immunol..

[10] Hou K., Wu Z.-X., Chen X.-Y., Wang J.-Q., Zhang D., Xiao C., Zhu D., Koya J.B., Wei L., Li J. (2022). Microbiota in health and diseases. Signal Transduct. Target. Ther..

[11] Di Tommaso N., Gasbarrini A., Ponziani F.R. (2021). Intestinal Barrier in Human Health and Disease. Int. J. Environ. Res. Public Health.

[12] Lin Q., Panchamukhi A., Li P., Shan W., Zhou H., Hou L., Chen W. (2021). *Malassezia* and *Staphylococcus* dominate scalp microbiome for seborrheic dermatitis. Bioprocess Biosyst. Eng..

[13] Szabó K., Bolla B.S., Erdei L., Balogh F., Kemény L. (2023). Are the Cutaneous Microbiota a Guardian of the Skin’s Physical Barrier? The Intricate Relationship between Skin Microbes and Barrier Integrity. Int. J. Mol. Sci..

[14] Weidinger S., Beck L.A., Bieber T., Kabashima K., Irvine A.D. (2018). Atopic dermatitis. Nat. Rev. Dis. Primer.

[15] Celoria V., Rosset F., Pala V., Dapavo P., Ribero S., Quaglino P., Mastorino L. (2023). The Skin Microbiome and Its Role in Psoriasis: A Review. Psoriasis.

[16] Ganju P., Nagpal S., Mohammed M.H., Nishal Kumar P., Pandey R., Natarajan V.T., Mande S.S., Gokhale R.S. (2016). Microbial community profiling shows dysbiosis in the lesional skin of Vitiligo subjects. Sci. Rep..

[17] Schell S.L., Schneider A.M., Nelson A.M. (2021). Yin and Yang: A disrupted skin microbiome and an aberrant host immune response in hidradenitis suppurativa. Exp. Dermatol..

[18] Pinto D., Sorbellini E., Marzani B., Rucco M., Giuliani G., Rinaldi F. (2019). Scalp bacterial shift in Alopecia areata. PLoS ONE.

[19] Huang C., Yi X., Long H., Zhang G., Wu H., Zhao M., Lu Q. (2020). Disordered cutaneous microbiota in systemic lupus erythematosus. J. Autoimmun..

[20] Savoia P., Azzimonti B., Rolla R., Zavattaro E. (2023). Role of the Microbiota in Skin Neoplasms: New Therapeutic Horizons. Microorganisms.

[21] Chen G., Chen Z.-M., Fan X.-Y., Jin Y.-L., Li X., Wu S.-R., Ge W.-W., Lv C.-H., Wang Y.-K., Chen J.-G. (2021). Gut-Brain-Skin Axis in Psoriasis: A Review. Dermatol. Ther..

[22] Mehta A.B., Nadkarni N.J., Patil S.P., Godse K.V., Gautam M., Agarwal S. (2016). Topical corticosteroids in dermatology. Indian J. Dermatol. Venereol. Leprol..

[23] Starbek Zorko M., Benko M., Rakuša M., Prunk Zdravković T. (2023). Evaluation of corticophobia in patients with atopic dermatitis and psoriasis using the TOPICOP© score. Acta Dermatovenerol. Alp. Pannonica Adriat..

[24] Yazici D., Ogulur I., Pat Y., Babayev H., Barletta E., Ardicli S., Bel Imam M., Huang M., Koch J., Li M. (2023). The epithelial barrier: The gateway to allergic, autoimmune, and metabolic diseases and chronic neuropsychiatric conditions. Semin. Immunol..

[25] Belkaid Y., Harrison O.J. (2017). Homeostatic Immunity and the Microbiota. Immunity.

[26] Yeshi K., Crayn D., Ritmejerytė E., Wangchuk P. (2022). Plant Secondary Metabolites Produced in Response to Abiotic Stresses Has Potential Application in Pharmaceutical Product Development. Molecules.

[27] Sangiovanni E., Dell’Agli M. (2021). Special Issue: Anti-Inflammatory Activity of Plant Polyphenols 2.0. Biomedicines.

[28] Rajha H.N., Paule A., Aragonès G., Barbosa M., Caddeo C., Debs E., Dinkova R., Eckert G.P., Fontana A., Gebrayel P. (2022). Recent Advances in Research on Polyphenols: Effects on Microbiota, Metabolism, and Health. Mol. Nutr. Food Res..

[29] Merecz-Sadowska A., Sitarek P., Zajdel K., Kucharska E., Kowalczyk T., Zajdel R. (2021). The Modulatory Influence of Plant-Derived Compounds on Human Keratinocyte Function. Int. J. Mol. Sci..

[30] Moldovan M.L., Carpa R., Fizeșan I., Vlase L., Bogdan C., Iurian S.M., Benedec D., Pop A. (2020). Phytochemical Profile and Biological Activities of Tendrils and Leaves Extracts from a Variety of *Vitis vinifera* L.. Antioxidants.

[31] Singh J., Rasane P., Kaur R., Kaur H., Garg R., Kaur S., Ercisli S., Choudhary R., Skrovankova S., Mlcek J. (2023). Valorization of grape (*Vitis vinifera*) leaves for bioactive compounds: Novel green extraction technologies and food-pharma applications. Front. Chem..

[32] Romani A., Campo M., Urciuoli S., Marrone G., Noce A., Bernini R. (2020). An Industrial and Sustainable Platform for the Production of Bioactive Micronized Powders and Extracts Enriched in Polyphenols From *Olea europaea* L. and *Vitis vinifera* L. Wastes. Front. Nutr..

[33] Baroi A.M., Popitiu M., Fierascu I., Sărdărescu I.-D., Fierascu R.C. (2022). Grapevine Wastes: A Rich Source of Antioxidants and Other Biologically Active Compounds. Antioxidants.

[34] Sharafan M., Malinowska M.A., Ekiert H., Kwaśniak B., Sikora E., Szopa A. (2023). *Vitis vinifera* (Vine Grape) as a Valuable Cosmetic Raw Material. Pharmaceutics.

[35] Sangiovanni E., Di Lorenzo C., Piazza S., Manzoni Y., Brunelli C., Fumagalli M., Magnavacca A., Martinelli G., Colombo F., Casiraghi A. (2019). *Vitis vinifera* L. Leaf Extract Inhibits In Vitro Mediators of Inflammation and Oxidative Stress Involved in Inflammatory-Based Skin Diseases. Antioxidants.

[36] Marabini L., Melzi G., Lolli F., Dell’Agli M., Piazza S., Sangiovanni E., Marinovich M. (2020). Effects of *Vitis vinifera* L. leaves extract on UV radiation damage in human keratinocytes (HaCaT). J. Photochem. Photobiol. B.

[37] Bogdan C., Pop A., Iurian S.M., Benedec D., Moldovan M.L. (2020). Research Advances in the Use of Bioactive Compounds from *Vitis vinifera* By-Products in Oral Care. Antioxidants.

[38] Piazza S., Fumagalli M., Khalilpour S., Martinelli G., Magnavacca A., Dell’Agli M., Sangiovanni E. (2020). A Review of the Potential Benefits of Plants Producing Berries in Skin Disorders. Antioxidants.

[39] Biniek K., Levi K., Dauskardt R.H. (2012). Solar UV radiation reduces the barrier function of human skin. Proc. Natl. Acad. Sci. USA.

[40] Passeron T., Krutmann J., Andersen M.L., Katta R., Zouboulis C.C. (2020). Clinical and biological impact of the exposome on the skin. J. Eur. Acad. Dermatol. Venereol. JEADV.

[41] Khmaladze I., Leonardi M., Fabre S., Messaraa C., Mavon A. (2020). The Skin Interactome: A Holistic “Genome-Microbiome-Exposome” Approach to Understand and Modulate Skin Health and Aging. Clin. Cosmet. Investig. Dermatol..

[42] Cecchi L., D’Amato G., Annesi-Maesano I. (2018). External exposome and allergic respiratory and skin diseases. J. Allergy Clin. Immunol..

[43] Prieux R., Eeman M., Rothen-Rutishauser B., Valacchi G. (2020). Mimicking cigarette smoke exposure to assess cutaneous toxicity. Toxicol. Vitr..

[44] Lima C., Falcão M.A.P., Rosa J.G.S., Disner G.R., Lopes-Ferreira M. (2022). Pesticides and Their Impairing Effects on Epithelial Barrier Integrity, Dysbiosis, Disruption of the AhR Signaling Pathway and Development of Immune-Mediated Inflammatory Diseases. Int. J. Mol. Sci..

[45] Belzer A., Parker E.R. (2023). Climate Change, Skin Health, and Dermatologic Disease: A Guide for the Dermatologist. Am. J. Clin. Dermatol..

[46] Aşkın Ö., Uzunçakmak T.K.Ü., Altunkalem N., Tüzün Y. (2021). Vitamin deficiencies/hypervitaminosis and the skin. Clin. Dermatol..

[47] Joshi M., Hiremath P., John J., Ranadive N., Nandakumar K., Mudgal J. (2023). Modulatory role of vitamins A, B3, C, D, and E on skin health, immunity, microbiome, and diseases. Pharmacol. Rep. PR.

[48] Kanda N., Hoashi T., Saeki H. (2019). The Roles of Sex Hormones in the Course of Atopic Dermatitis. Int. J. Mol. Sci..

[49] Gratton R., Del Vecchio C., Zupin L., Crovella S. (2022). Unraveling the Role of Sex Hormones on Keratinocyte Functions in Human Inflammatory Skin Diseases. Int. J. Mol. Sci..

[50] Orion E., Wolf R. (2012). Psychological stress and epidermal barrier function. Clin. Dermatol..

[51] Chen Y., Lyga J. (2014). Brain-Skin Connection: Stress, Inflammation and Skin Aging. Inflamm. Allergy Drug Targets.

[52] Choe S.J., Kim D., Kim E.J., Ahn J.-S., Choi E.-J., Son E.D., Lee T.R., Choi E.H. (2018). Psychological Stress Deteriorates Skin Barrier Function by Activating 11β-Hydroxysteroid Dehydrogenase 1 and the HPA Axis. Sci. Rep..

[53] Zhang H., Wang M., Zhao X., Wang Y., Chen X., Su J. (2024). Role of stress in skin diseases: A neuroendocrine-immune interaction view. Brain Behav. Immun..

[54] Li W., Wang Z., Cao J., Dong Y., Chen Y. (2023). Melatonin improves skin barrier damage caused by sleep restriction through gut microbiota. J. Pineal Res..

[55] Parrish A.R. (2017). The impact of aging on epithelial barriers. Tissue Barriers.

[56] Agrawal R., Hu A., Bollag W.B. (2023). The Skin and Inflamm-Aging. Biology.

[57] Lee H.-J., Kim M. (2022). Skin Barrier Function and the Microbiome. Int. J. Mol. Sci..

[58] Neveu V., Nicolas G., Amara A., Salek R.M., Scalbert A. (2023). The human microbial exposome: Expanding the Exposome-Explorer database with gut microbial metabolites. Sci. Rep..

[59] Irvine A.D., McLean W.H.I., Leung D.Y.M. (2011). Filaggrin mutations associated with skin and allergic diseases. N. Engl. J. Med..

[60] Schleimer R.P., Berdnikovs S. (2017). Etiology of epithelial barrier dysfunction in patients with type 2 inflammatory diseases. J. Allergy Clin. Immunol..

[61] Kucuksezer U.C., Ozdemir C., Yazici D., Pat Y., Mitamura Y., Li M., Sun N., D’Avino P., Bu X., Zhu X. (2023). The epithelial barrier theory: Development and exacerbation of allergic and other chronic inflammatory diseases. Asia Pac. Allergy.

[62] Wagner R.N., Piñón Hofbauer J., Wally V., Kofler B., Schmuth M., De Rosa L., De Luca M., Bauer J.W. (2021). Epigenetic and metabolic regulation of epidermal homeostasis. Exp. Dermatol..

[63] Kimball A.B. (2008). Skin differences, needs, and disorders across global populations. J. Investig. Dermatol. Symp. Proc..

[64] Andersen L.K., Davis M.D.P. (2016). Sex differences in the incidence of skin and skin-related diseases in Olmsted County, Minnesota, United States, and a comparison with other rates published worldwide. Int. J. Dermatol..

[65] Rahrovan S., Fanian F., Mehryan P., Humbert P., Firooz A. (2018). Male versus female skin: What dermatologists and cosmeticians should know. Int. J. Womens Dermatol..

[66] Lagacé F., D’Aguanno K., Prosty C., Laverde-Saad A., Cattelan L., Ouchene L., Oliel S., Genest G., Doiron P., Richer V. (2023). The Role of Sex and Gender in Dermatology—From Pathogenesis to Clinical Implications. J. Cutan. Med. Surg..

[67] Cantwell M.I., Hong G., Albornoz K., Berlanga M. (2022). Fresh grapevine (*Vitis vinifera* L.) leaves: Postharvest biology and handling recommendations. Sci. Hortic..

[68] Vinci G., Prencipe S.A., Abbafati A., Filippi M. (2022). Environmental Impact Assessment of an Organic Wine Production in Central Italy: Case Study from Lazio. Sustainability.

[69] Wong M.C., Hendrikse S.I.S., Sherrell P.C., Ellis A.V. (2020). Grapevine waste in sustainable hybrid particleboard production. Waste Manag..

[70] Ahmad B., Yadav V., Yadav A., Rahman M.U., Yuan W.Z., Li Z., Wang X. (2020). Integrated biorefinery approach to valorize winery waste: A review from waste to energy perspectives. Sci. Total Environ..

[71] Lacerda D.D.S., Santos C.F., Oliveira A.S., Zimmermann R., Schneider R., Agostini F., Dani C., Funchal C., Gomez R. (2014). Antioxidant and hepatoprotective effects of an organic grapevine leaf (*Vitis labrusca* L.) extract in diabetic rats. RSC Adv..

[72] Gonçalves D.A., González A., Roupar D., Teixeira J.A., Nobre C. (2023). How prebiotics have been produced from agro-industrial waste: An overview of the enzymatic technologies applied and the models used to validate their health claims. Trends Food Sci. Technol..

[73] Chiocchio I., Mandrone M., Tomasi P., Marincich L., Poli F. (2021). Plant Secondary Metabolites: An Opportunity for Circular Economy. Molecules.

[74] Kortekamp A. (2006). Expression analysis of defence-related genes in grapevine leaves after inoculation with a host and a non-host pathogen. Plant Physiol. Biochem. PPB.

[75] Garrido J., Borges F. (2013). Wine and grape polyphenols—A chemical perspective. Food Res. Int..

[76] Guerriero G., Berni R., Muñoz-Sanchez J.A., Apone F., Abdel-Salam E.M., Qahtan A.A., Alatar A.A., Cantini C., Cai G., Hausman J.-F. (2018). Production of Plant Secondary Metabolites: Examples, Tips and Suggestions for Biotechnologists. Genes.

[77] Fiume M.M., Bergfeld W.F., Belsito D.V., Hill R.A., Klaassen C.D., Liebler D.C., Marks J.G., Shank R.C., Slaga T.J., Snyder P.W. (2014). Safety assessment of *Vitis vinifera* (grape)-derived ingredients as used in cosmetics. Int. J. Toxicol..

[78] Tabeshpour J., Mehri S., Shaebani Behbahani F., Hosseinzadeh H. (2018). Protective effects of *Vitis vinifera* (grapes) and one of its biologically active constituents, resveratrol, against natural and chemical toxicities: A comprehensive review. Phytother. Res..

[79] Katalinić V., Mozina S.S., Generalic I., Skroza D., Ljubenkov I., Klancnik A. (2013). Phenolic Profile, Antioxidant Capacity, and Antimicrobial Activity of Leaf Extracts from Six *Vitis vinifera* L. Varieties. Int. J. Food Prop..

[80] Anđelković M., Radovanović B., Anđelković A.M., Radovanović V. (2015). Phenolic Compounds and Bioactivity of Healthy and Infected Grapevine Leaf Extracts from Red Varieties Merlot and Vranac (*Vitis vinifera* L.). Plant Foods Hum. Nutr..

[81] Páscoa R.N.M.J. (2018). In Situ Visible and Near-Infrared Spectroscopy Applied to Vineyards as a Tool for Precision Viticulture. Comprehensive Analytical Chemistry.

[82] Gülcü M., Ghafoor K., Al-Juhaimi F., Özcan M.M., Uslu N., Babiker E.E., Ahmed I.A.M., Azmi I.U. (2020). Effect of grape (*Vitis vinifera* L.) varieties and harvest periods on bioactive compounds, antioxidant activity, phenolic composition, mineral contents, and fatty acid compositions of Vitis leave and oils. J. Food Process. Preserv..

[83] Goufo P., Singh R.K., Cortez I. (2020). A Reference List of Phenolic Compounds (Including Stilbenes) in Grapevine (*Vitis vinifera* L.) Roots, Woods, Canes, Stems, and Leaves. Antioxidants.

[84] Yue X., Zhao Y., Ma X., Jiao X., Fang Y., Zhang Z., Ju Y. (2021). Effects of leaf removal on the accumulation of anthocyanins and the expression of anthocyanin biosynthetic genes in Cabernet Sauvignon (*Vitis vinifera* L.) grapes. J. Sci. Food Agric..

[85] Harb J., Alseekh S., Tohge T., Fernie A.R. (2015). Profiling of primary metabolites and flavonols in leaves of two table grape varieties collected from semiarid and temperate regions. Phytochemistry.

[86] Cui Z.-H., Bi W.-L., Hao X.-Y., Li P.-M., Duan Y., Walker M.A., Xu Y., Wang Q.-C. (2017). Drought Stress Enhances Up-Regulation of Anthocyanin Biosynthesis in Grapevine leafroll-associated virus 3-Infected in vitro Grapevine (*Vitis vinifera*) Leaves. Plant Dis..

[87] Tartaglione L., Gambuti A., De Cicco P., Ercolano G., Ianaro A., Taglialatela-Scafati O., Moio L., Forino M. (2018). NMR-based phytochemical analysis of *Vitis vinifera* cv Falanghina leaves. Characterization of a previously undescribed biflavonoid with antiproliferative activity. Fitoterapia.

[88] Lima M.R.M., Felgueiras M.L., Cunha A., Chicau G., Ferreres F., Dias A.C.P. (2017). Differential phenolic production in leaves of *Vitis vinifera* cv. Alvarinho affected with esca disease. Plant Physiol. Biochem. PPB.

[89] Djemaa-Landri K., Hamri-Zeghichi S., Valls J., Cluzet S., Tristan R., Boulahbal N., Kadri N., Madani K. (2020). Phenolic content and antioxidant activities of *Vitis vinifera* L. leaf extracts obtained by conventional solvent and microwave-assisted extractions. J. Food Meas. Charact..

[90] Jediyi H., Naamani K., Ait Elkoch A., Dihazi A., Lemjiber N. (2020). A comparative study of phenols composition, antioxidant, and antifungal potency of leaves extract from five Moroccan *Vitis vinifera* L. varieties. J. Food Saf..

[91] Pantelić M.M., Zagorac D.Č.D., Ćirić I.Ž., Pergal M.V., Relić D.J., Todić S.R., Natić M.M. (2017). Phenolic profiles, antioxidant activity and minerals in leaves of different grapevine varieties grown in Serbia. J. Food Compos. Anal..

[92] Farhadi K., Esmaeilzadeh F., Hatami M., Forough M., Molaie R. (2016). Determination of phenolic compounds content and antioxidant activity in skin, pulp, seed, cane and leaf of five native grape cultivars in West Azerbaijan province, Iran. Food Chem..

[93] Aouey B., Samet A.M., Fetoui H., Simmonds M.S.J., Bouaziz M. (2016). Anti-oxidant, anti-inflammatory, analgesic and antipyretic activities of grapevine leaf extract (*Vitis vinifera)* in mice and identification of its active constituents by LC–MS/MS analyses. Biomed. Pharmacother..

[94] Vrhovsek U., Malacarne G., Masuero D., Zulini L., Guella G., Stefanini M., Velasco R., Mattivi F. (2012). Profiling and accurate quantification of *trans*-resveratrol, *trans*-piceid, *trans*-pterostilbene and 11 viniferins induced by *Plasmopara viticola* in partially resistant grapevine leaves. Aust. J. Grape Wine Res..

[95] Šuković D., Knežević B., Gašić U., Sredojević M., Ćirić I., Todić S., Mutić J., Tešić Ž. (2020). Phenolic Profiles of Leaves, Grapes and Wine of Grapevine Variety Vranac (*Vitis vinifera* L.) from Montenegro. Foods.

[96] Mattivi F., Vrhovsek U., Malacarne G., Masuero D., Zulini L., Stefanini M., Moser C., Velasco R., Guella G. (2011). Profiling of Resveratrol Oligomers, Important Stress Metabolites, Accumulating in the Leaves of Hybrid *Vitis vinifera* (Merzling × Teroldego) Genotypes Infected with *Plasmopara viticola*. J. Agric. Food Chem..

[97] Rätsep R., Karp K., Maante-Kuljus M., Aluvee A., Bhat R. (2020). Polyphenols and Resveratrol from Discarded Leaf Biomass of Grapevine (*Vitis* sp.): Effect of Cultivar and Viticultural Practices in Estonia. Agriculture.

[98] Sun H., Lin Q., Wei W., Qin G. (2018). Ultrasound-assisted extraction of resveratrol from grape leaves and its purification on mesoporous carbon. Food Sci. Biotechnol..

[99] Dadáková K., Jurasová L., Kašparovský T., Průšová B., Baroň M., Sochor J. (2021). Origin of Wine Lignans. Plant Foods Hum. Nutr..

[100] Tylewicz U., Nowacka M., Martín-García B., Wiktor A., Gómez Caravaca A.M., Galanakis C.M. (2018). 5—Target sources of polyphenols in different food products and their processing by-products. Polyphenols: Properties, Recovery, and Applications.

[101] Li X., Liu C., Li Y., Yuan K., Zhang W., Cai D., Peng Z., Hu Y., Sun J., Bai W. (2023). Bioactivity and application of anthocyanins in skin protection and cosmetics: An extension as a functional pigment. Phytochem. Rev..

[102] Aron P.M., Kennedy J.A. (2008). Flavan-3-ols: Nature, occurrence and biological activity. Mol. Nutr. Food Res..

[103] Gervasi T., Calderaro A., Barreca D., Tellone E., Trombetta D., Ficarra S., Smeriglio A., Mandalari G., Gattuso G. (2022). Biotechnological Applications and Health-Promoting Properties of Flavonols: An Updated View. Int. J. Mol. Sci..

[104] Vattem D.A., Shetty K. (2005). Biological Functionality of Ellagic Acid: A Review. J. Food Biochem..

[105] Teka T., Zhang L., Ge X., Li Y., Han L., Yan X. (2022). Stilbenes: Source plants, chemistry, biosynthesis, pharmacology, application and problems related to their clinical Application-A comprehensive review. Phytochemistry.

[106] Kasperkiewicz K., Erkiert-Polguj A., Budzisz E. (2016). Sunscreening and Photosensitizing Properties of Coumarins and their Derivatives. Lett. Drug Des. Discov..

[107] Garg S.S., Gupta J., Sharma S., Sahu D. (2020). An insight into the therapeutic applications of coumarin compounds and their mechanisms of action. Eur. J. Pharm. Sci..

[108] Osmakov D.I., Kalinovskii A.P., Belozerova O.A., Andreev Y.A., Kozlov S.A. (2022). Lignans as Pharmacological Agents in Disorders Related to Oxidative Stress and Inflammation: Chemical Synthesis Approaches and Biological Activities. Int. J. Mol. Sci..

[109] World Health Organization WHO Traditional Medicine Strategy: 2014–2023. http://web.archive.org/web/20220912054716/https://apps.who.int/iris/handle/10665/92455.

[110] Yuan H., Ma Q., Ye L., Piao G. (2016). The Traditional Medicine and Modern Medicine from Natural Products. Molecules.

[111] Chen H., Su Z., Pan X., Zheng X., Li H., Ye Z., Tang B., Lu Y., Zheng G., Lu C. (2023). Phytochemicals: Targeting autophagy to treat psoriasis. Phytomedicine.

[112] Fernandes F., Ramalhosa E., Pires P., Verdial J., Valentão P., Andrade P., Bento A., Pereira J.A. (2013). *Vitis vinifera* leaves towards bioactivity. Ind. Crops Prod..

[113] Ribeiro A.S., Estanqueiro M., Oliveira M.B., Sousa Lobo J.M. (2015). Main Benefits and Applicability of Plant Extracts in Skin Care Products. Cosmetics.

[114] Khan M.K., Hassan S., Paniwnyk L., Li Y., Chemat F. (2019). Polyphenols as Natural Antioxidants: Sources, Extraction and Applications in Food, Cosmetics and Drugs. Plant Based “Green Chemistry 2.0”.

[115] Castro M.L., Ferreira J.P., Pintado M., Ramos O.L., Borges S., Baptista-Silva S. (2023). Grape By-Products in Sustainable Cosmetics: Nanoencapsulation and Market Trends. Appl. Sci..

[116] De Almeida C.V., Antiga E., Lulli M. (2023). Oral and Topical Probiotics and Postbiotics in Skincare and Dermatological Therapy: A Concise Review. Microorganisms.

[117] Maia M., Ferreira A.E.N., Laureano G., Marques A.P., Torres V.M., Silva A.B., Matos A.R., Cordeiro C., Figueiredo A., Silva M.S. (2019). *Vitis vinifera* ‘Pinot noir’ leaves as a source of bioactive nutraceutical compounds. Food Funct..

[118] Filocamo A., Bisignano C., Mandalari G., Navarra M. (2015). In Vitro Antimicrobial Activity and Effect on Biofilm Production of a White Grape Juice (*Vitis vinifera*) Extract. Evid.-Based Complement. Altern. Med. ECAM.

[119] Cefali L.C., Ataide J.A., Sousa I.M.d.O., Figueiredo M.C., Ruiz A.L.T.G., Foglio M.A., Mazzola P.G. (2020). In vitro solar protection factor, antioxidant activity, and stability of a topical formulation containing Benitaka grape (*Vitis vinifera* L.) peel extract. Nat. Prod. Res..

[120] Harris-Tryon T.A., Grice E.A. (2022). Microbiota and maintenance of skin barrier function. Science.

[121] Lefèvre-Utile A., Braun C., Haftek M., Aubin F. (2021). Five Functional Aspects of the Epidermal Barrier. Int. J. Mol. Sci..

[122] Turnbaugh P.J., Ley R.E., Hamady M., Fraser-Liggett C.M., Knight R., Gordon J.I. (2007). The human microbiome project. Nature.

[123] Sanford J.A., Gallo R.L. (2013). Functions of the skin microbiota in health and disease. Semin. Immunol..

[124] Callewaert C., Ravard Helffer K., Lebaron P. (2020). Skin Microbiome and its Interplay with the Environment. Am. J. Clin. Dermatol..

[125] Luna P.C. (2020). Skin Microbiome as Years Go By. Am. J. Clin. Dermatol..

[126] Azzimonti B., Ballacchino C., Zanetta P., Cucci M.A., Monge C., Grattarola M., Dianzani C., Barrera G., Pizzimenti S. (2023). Microbiota, Oxidative Stress, and Skin Cancer: An Unexpected Triangle. Antioxidants.

[127] Pinto D., Ciardiello T., Franzoni M., Pasini F., Giuliani G., Rinaldi F. (2021). Effect of commonly used cosmetic preservatives on skin resident microflora dynamics. Sci. Rep..

[128] Zanetta P., Ballacchino C., Squarzanti D.F., Amoruso A., Pane M., Azzimonti B. (2023). *Lactobacillus johnsonii* LJO02 (DSM 33828) Cell-Free Supernatant and Vitamin D Improve Wound Healing and Reduce Interleukin-6 Production in *Staphylococcus aureus*-Infected Human Keratinocytes. Pharmaceutics.

[129] Cogen A.L., Nizet V., Gallo R.L. (2008). Skin microbiota: A source of disease or defence?. Br. J. Dermatol..

[130] Betsi G.I., Papadavid E., Falagas M.E. (2008). Probiotics for the treatment or prevention of atopic dermatitis: A review of the evidence from randomized controlled trials. Am. J. Clin. Dermatol..

[131] Rook G.a.W., Adams V., Hunt J., Palmer R., Martinelli R., Brunet L.R. (2004). Mycobacteria and other environmental organisms as immunomodulators for immunoregulatory disorders. Springer Semin. Immunopathol..

[132] Haahtela T., Holgate S., Pawankar R., Akdis C.A., Benjaponpitak S., Caraballo L., Demain J., Portnoy J., von Hertzen L. (2013). WAO Special Committee on Climate Change and Biodiversity The biodiversity hypothesis and allergic disease: World allergy organization position statement. World Allergy Organ. J..

[133] Tsuge M., Ikeda M., Matsumoto N., Yorifuji T., Tsukahara H. (2021). Current Insights into Atopic March. Children.

[134] Kim K., Jang H., Kim E., Kim H., Sung G.Y. (2023). Recent advances in understanding the role of the skin microbiome in the treatment of atopic dermatitis. Exp. Dermatol..

[135] Wikramanayake T.C., Borda L.J., Miteva M., Paus R. (2019). Seborrheic dermatitis-Looking beyond *Malassezia*. Exp. Dermatol..

[136] Jackson J.M., Alexis A., Zirwas M., Taylor S. (2024). Unmet needs for patients with seborrheic dermatitis. J. Am. Acad. Dermatol..

[137] Huang C., Zhuo F., Han B., Li W., Jiang B., Zhang K., Jian X., Chen Z., Li H., Huang H. (2023). The updates and implications of cutaneous microbiota in acne. Cell Biosci..

[138] Dreno B., Dekio I., Baldwin H., Demessant A.L., Dagnelie M.-A., Khammari A., Corvec S. (2024). Acne microbiome: From phyla to phylotypes. J. Eur. Acad. Dermatol. Venereol..

[139] Świerczewska Z., Lewandowski M., Surowiecka A., Barańska-Rybak W. (2022). Microbiome in Hidradenitis Suppurativa-What We Know and Where We Are Heading. Int. J. Mol. Sci..

[140] Rosi E., Guerra P., Silvi G., Nunziati G., Scandagli I., Di Cesare A., Prignano F. (2023). Consistency of Bacterial Triggers in the Pathogenesis of Hidradenitis Suppurativa. Vaccines.

[141] Rendon A., Schäkel K. (2019). Psoriasis Pathogenesis and Treatment. Int. J. Mol. Sci..

[142] Benhadou F., Mintoff D., Schnebert B., Thio H.B. (2018). Psoriasis and Microbiota: A Systematic Review. Diseases.

[143] Soleymani S., Iranpanah A., Najafi F., Belwal T., Ramola S., Abbasabadi Z., Momtaz S., Farzaei M.H. (2019). Implications of grape extract and its nanoformulated bioactive agent resveratrol against skin disorders. Arch. Dermatol. Res..

[144] Wen S., Zhang J., Yang B., Elias P.M., Man M.-Q. (2020). Role of Resveratrol in Regulating Cutaneous Functions. Evid.-Based Complement. Altern. Med. ECAM.

[145] Wang J., Chen W.-D., Wang Y.-D. (2020). The Relationship Between Gut Microbiota and Inflammatory Diseases: The Role of Macrophages. Front. Microbiol..

[146] Marko M., Pawliczak R. (2023). Resveratrol and Its Derivatives in Inflammatory Skin Disorders-Atopic Dermatitis and Psoriasis: A Review. Antioxidants.

[147] Pennesi C.M., Neely J., Marks A.G., Basak S.A. (2017). Use of Isoquercetin in the Treatment of Prurigo Nodularis. J. Drugs Dermatol..

[148] Fabbrocini G., Cameli N., Lorenzi S., De Padova M.P., Marasca C., Izzo R., Monfrecola G. (2014). A dietary supplement to reduce side effects of oral isotretinoin therapy in acne patients. G Ital. Dermatol. Venereol..

[149] Singh C.K., Mintie C.A., Ndiaye M.A., Chhabra G., Roy S., Sullivan R., Longley B.J., Schieke S.M., Ahmad N. (2022). Protective effects of dietary grape against atopic dermatitis-like skin lesions in NC/NgaTndCrlj mice. Front. Immunol..

[150] Bangash Y., Saleem A., Akhtar M.F., Anwar F., Akhtar B., Sharif A., Khan M.I., Khan A. (2023). Pterostilbene reduces the progression of atopic dermatitis via modulating inflammatory and oxidative stress biomarkers in mice. Inflammopharmacology.

[151] Nelson K., Lyles J.T., Li T., Saitta A., Addie-Noye E., Tyler P., Quave C.L. (2016). Anti-Acne Activity of Italian Medicinal Plants Used for Skin Infection. Front. Pharmacol..

[152] Lim H.-J., Kang S.-H., Song Y.-J., Jeon Y.-D., Jin J.-S. (2021). Inhibitory Effect of Quercetin on *Propionibacterium acnes*-induced Skin Inflammation. Int. Immunopharmacol..

[153] Witte K., Wolk K., Witte-Händel E., Krause T., Kokolakis G., Sabat R. (2023). Targeting Metabolic Syndrome in Hidradenitis Suppurativa by Phytochemicals as a Potential Complementary Therapeutic Strategy. Nutrients.

[154] Simonetti G., D’Auria F.D., Mulinacci N., Innocenti M., Antonacci D., Angiolella L., Santamaria A.R., Valletta A., Donati L., Pasqua G. (2017). Anti-Dermatophyte and Anti-*Malassezia* Activity of Extracts Rich in Polymeric Flavan-3-ols Obtained from *Vitis vinifera* Seeds. Phytother. Res. PTR.

[155] Mustarichie R., Rostinawati T., Pitaloka D.A.E., Saptarini N.M., Iskandar Y. (2022). Herbal Therapy for the Treatment of Seborrhea Dermatitis. Clin. Cosmet. Investig. Dermatol..

[156] Takahashi T., Kamiya T., Hasegawa A., Yokoo Y. (1999). Procyanidin oligomers selectively and intensively promote proliferation of mouse hair epithelial cells in vitro and activate hair follicle growth in vivo. J. Investig. Dermatol..

[157] Lombardo G., Melzi G., Indino S., Piazza S., Sangiovanni E., Baruffaldi Preis F., Marabini L., Donetti E. (2022). Keratin 17 as a Marker of UVB-Induced Stress in Human Epidermis and Modulation by *Vitis vinifera* Extract. Cells Tissues Organs.

[158] Letsiou S., Kapazoglou A., Tsaftaris A., Spanidi E., Gardikis K. (2020). Transcriptional and epigenetic effects of *Vitis vinifera* L. leaf extract on UV-stressed human dermal fibroblasts. Mol. Biol. Rep..

[159] Hamada H., Shimoda K., Horio Y., Onoa T., Hosoda R., Nakayama N., Uranod K. (2017). Pterostilbene and Its Glucoside Induce Type XVII Collagen Expression. Nat. Prod. Commun..

[160] Liu Y., Ho C., Wen D., Sun J., Huang L., Gao Y., Li Q., Zhang Y. (2022). Targeting the stem cell niche: Role of collagen XVII in skin aging and wound repair. Theranostics.

[161] Makarewicz M., Drożdż I., Tarko T., Duda-Chodak A. (2021). The Interactions between Polyphenols and Microorganisms, Especially Gut Microbiota. Antioxidants.

[162] Milutinović M., Dimitrijević-Branković S., Rajilić-Stojanović M. (2021). Plant Extracts Rich in Polyphenols as Potent Modulators in the Growth of Probiotic and Pathogenic Intestinal Microorganisms. Front. Nutr..

[163] Rodríguez-Daza M.C., Pulido-Mateos E.C., Lupien-Meilleur J., Guyonnet D., Desjardins Y., Roy D. (2021). Polyphenol-Mediated Gut Microbiota Modulation: Toward Prebiotics and Further. Front. Nutr..

[164] Fraga C.G., Croft K.D., Kennedy D.O., Tomás-Barberán F.A. (2019). The effects of polyphenols and other bioactives on human health. Food Funct..

[165] Gibson G.R., Hutkins R., Sanders M.E., Prescott S.L., Reimer R.A., Salminen S.J., Scott K., Stanton C., Swanson K.S., Cani P.D. (2017). Expert consensus document: The International Scientific Association for Probiotics and Prebiotics (ISAPP) consensus statement on the definition and scope of prebiotics. Nat. Rev. Gastroenterol. Hepatol..

[166] Corrêa T.A.F., Rogero M.M., Hassimotto N.M.A., Lajolo F.M. (2019). The Two-Way Polyphenols-Microbiota Interactions and Their Effects on Obesity and Related Metabolic Diseases. Front. Nutr..

[167] Westfall S., Pasinetti G.M. (2019). The Gut Microbiota Links Dietary Polyphenols with Management of Psychiatric Mood Disorders. Front. Neurosci..

[168] Chakkalakal M., Nadora D., Gahoonia N., Dumont A., Burney W., Pan A., Chambers C.J., Sivamani R.K. (2022). Prospective Randomized Double-Blind Placebo-Controlled Study of Oral Pomegranate Extract on Skin Wrinkles, Biophysical Features, and the Gut-Skin Axis. J. Clin. Med..

[169] Xia E.-Q., Deng G.-F., Guo Y.-J., Li H.-B. (2010). Biological activities of polyphenols from grapes. Int. J. Mol. Sci..

[170] Cheng V.J., Bekhit A.E.-D.A., McConnell M., Mros S., Zhao J. (2012). Effect of extraction solvent, waste fraction and grape variety on the antimicrobial and antioxidant activities of extracts from wine residue from cool climate. Food Chem..

[171] Krasteva D., Ivanov Y., Chengolova Z., Godjevargova T. (2023). Antimicrobial Potential, Antioxidant Activity, and Phenolic Content of Grape Seed Extracts from Four Grape Varieties. Microorganisms.

[172] Pozzo L., Grande T., Raffaelli A., Longo V., Weidner S., Amarowicz R., Karamać M. (2023). Characterization of Antioxidant and Antimicrobial Activity and Phenolic Compound Profile of Extracts from Seeds of Different *Vitis* Species. Molecules.

[173] Daglia M. (2012). Polyphenols as antimicrobial agents. Curr. Opin. Biotechnol..

[174] Patangia D.V., Anthony Ryan C., Dempsey E., Paul Ross R., Stanton C. (2022). Impact of antibiotics on the human microbiome and consequences for host health. MicrobiologyOpen.

[175] Al Bander Z., Nitert M.D., Mousa A., Naderpoor N. (2020). The Gut Microbiota and Inflammation: An Overview. Int. J. Environ. Res. Public Health.

[176] Shabbir U., Tyagi A., Elahi F., Aloo S.O., Oh D.-H. (2021). The Potential Role of Polyphenols in Oxidative Stress and Inflammation Induced by Gut Microbiota in Alzheimer’s Disease. Antioxidants.

[177] Sun M., Deng Y., Cao X., Xiao L., Ding Q., Luo F., Huang P., Gao Y., Liu M., Zhao H. (2022). Effects of Natural Polyphenols on Skin and Hair Health: A Review. Molecules.

[178] Orhan D., Orhan N., Özçelik B., Ergun F. (2009). Biological Screening of *Vitis vinifera* L. Leaf Fractions. Turk. J. Biol..

[179] Ceyhan Guvensen N., Keskin D., Zorlu Z., Ugur A. (2012). In-vitro antimicrobial activities of different extracts of grapevine leaves (*Vitis vinifera* L.) from West Anatolia against some pathogenic microorganisms. J. Pure Appl. Microbiol..

[180] Leal C., Santos R.A., Pinto R., Queiroz M., Rodrigues M., José Saavedra M., Barros A., Gouvinhas I. (2020). Recovery of bioactive compounds from white grape (*Vitis vinifera* L.) stems as potential antimicrobial agents for human health. Saudi J. Biol. Sci..

[181] Radulescu C., Buruleanu L.C., Nicolescu C.M., Olteanu R.L., Bumbac M., Holban G.C., Simal-Gandara J. (2020). Phytochemical Profiles, Antioxidant and Antibacterial Activities of Grape (*Vitis vinifera* L.) Seeds and Skin from Organic and Conventional Vineyards. Plants.

[182] Oskay M., Oskay D. (2007). Antimicrobial Screening of Some Turkish Medicinal Plants. Pharm. Biol..

[183] Katalinić V., Možina S.S., Skroza D., Generalić I., Abramovič H., Miloš M., Ljubenkov I., Piskernik S., Pezo I., Terpinc P. (2010). Polyphenolic profile, antioxidant properties and antimicrobial activity of grape skin extracts of 14 *Vitis vinifera* varieties grown in Dalmatia (Croatia). Food Chem..

[184] Felhi S., Baccouch N., Ben Salah H., Smaoui S., Allouche N., Gharsallah N., Kadri A. (2016). Nutritional constituents, phytochemical profiles, in vitro antioxidant and antimicrobial properties, and gas chromatography-mass spectrometry analysis of various solvent extracts from grape seeds (*Vitis vinifera* L.). Food Sci. Biotechnol..

[185] Brochado A.R., Telzerow A., Bobonis J., Banzhaf M., Mateus A., Selkrig J., Huth E., Bassler S., Zamarreño Beas J., Zietek M. (2018). Species-specific activity of antibacterial drug combinations. Nature.

[186] Sun J., Rutherford S.T., Silhavy T.J., Huang K.C. (2022). Physical properties of the bacterial outer membrane. Nat. Rev. Microbiol..

[187] López C., Ayala J.A., Bonomo R.A., González L.J., Vila A.J. (2019). Protein determinants of dissemination and host specificity of metallo-β-lactamases. Nat. Commun..

[188] Matilla-Cuenca L., Gil C., Cuesta S., Rapún-Araiz B., Žiemytė M., Mira A., Lasa I., Valle J. (2020). Antibiofilm activity of flavonoids on staphylococcal biofilms through targeting BAP amyloids. Sci. Rep..

[189] Jensen G.S., Cruickshank D., Hamilton D.E. (2023). Disruption of Established Bacterial and Fungal Biofilms by a Blend of Enzymes and Botanical Extracts. J. Microbiol. Biotechnol..

[190] Kolouchová I., Maťátková O., Paldrychová M., Kodeš Z., Kvasničková E., Sigler K., Čejková A., Šmidrkal J., Demnerová K., Masák J. (2018). Resveratrol, pterostilbene, and baicalein: Plant-derived anti-biofilm agents. Folia Microbiol..

[191] Akinwumi B.C., Bordun K.-A.M., Anderson H.D. (2018). Biological Activities of Stilbenoids. Int. J. Mol. Sci..

[192] Mattio L.M., Dallavalle S., Musso L., Filardi R., Franzetti L., Pellegrino L., D’Incecco P., Mora D., Pinto A., Arioli S. (2019). Antimicrobial activity of resveratrol-derived monomers and dimers against foodborne pathogens. Sci. Rep..

[193] Schmidlin L., Poutaraud A., Claudel P., Mestre P., Prado E., Santos-Rosa M., Wiedemann-Merdinoglu S., Karst F., Merdinoglu D., Hugueney P. (2008). A stress-inducible resveratrol O-methyltransferase involved in the biosynthesis of pterostilbene in grapevine. Plant Physiol..

[194] Lim Y.R.I., Preshaw P.M., Lim L.P., Ong M.M.A., Lin H.-S., Tan K.S. (2020). Pterostilbene complexed with cyclodextrin exerts antimicrobial and anti-inflammatory effects. Sci. Rep..

[195] Coenye T., Brackman G., Rigole P., De Witte E., Honraet K., Rossel B., Nelis H.J. (2012). Eradication of *Propionibacterium acnes* biofilms by plant extracts and putative identification of icariin, resveratrol and salidroside as active compounds. Phytomedicine Int. J. Phytother. Phytopharm..

[196] Abbott C., Grout E., Morris T., Brown H.L. (2022). *Cutibacterium acnes* biofilm forming clinical isolates modify the formation and structure of *Staphylococcus aureus* biofilms, increasing their susceptibility to antibiotics. Anaerobe.

[197] Tomic-Canic M., Burgess J.L., O’Neill K.E., Strbo N., Pastar I. (2020). Skin Microbiota and its Interplay with Wound Healing. Am. J. Clin. Dermatol..

[198] Vestby L.K., Grønseth T., Simm R., Nesse L.L. (2020). Bacterial Biofilm and its Role in the Pathogenesis of Disease. Antibiotics.

[199] Yang S.-C., Tseng C.-H., Wang P.-W., Lu P.-L., Weng Y.-H., Yen F.-L., Fang J.-Y. (2017). Pterostilbene, a Methoxylated Resveratrol Derivative, Efficiently Eradicates Planktonic, Biofilm, and Intracellular MRSA by Topical Application. Front. Microbiol..

[200] Vaňková E., Paldrychová M., Kašparová P., Lokočová K., Kodeš Z., Maťátková O., Kolouchová I., Masák J. (2020). Natural antioxidant pterostilbene as an effective antibiofilm agent, particularly for gram-positive cocci. World J. Microbiol. Biotechnol..

[201] Zannella C., Giugliano R., Chianese A., Buonocore C., Vitale G.A., Sanna G., Sarno F., Manzin A., Nebbioso A., Termolino P. (2021). Antiviral Activity of *Vitis vinifera* Leaf Extract against SARS-CoV-2 and HSV-1. Viruses.

[202] Berardi V., Ricci F., Castelli M., Galati G., Risuleo G. (2009). Resveratrol exhibits a strong cytotoxic activity in cultured cells and has an antiviral action against polyomavirus: Potential clinical use. J. Exp. Clin. Cancer Res. CR.

[203] Friedman M. (2014). Antibacterial, antiviral, and antifungal properties of wines and winery byproducts in relation to their flavonoid content. J. Agric. Food Chem..

[204] Lin Y.-S., Chen H.-J., Huang J.-P., Lee P.-C., Tsai C.-R., Hsu T.-F., Huang W.-Y. (2017). Kinetics of Tyrosinase Inhibitory Activity Using *Vitis vinifera* Leaf Extracts. BioMed Res. Int..

[205] Sil A., Panigrahi A., Chandra A., Pramanik J.D. (2022). “COVID nose”—A unique post-COVID pigmentary sequelae reminiscing Chik sign: A descriptive case series. J. Eur. Acad. Dermatol. Venereol. JEADV.

[206] Ma Y., Madupu R., Karaoz U., Nossa C.W., Yang L., Yooseph S., Yachimski P.S., Brodie E.L., Nelson K.E., Pei Z. (2014). Human Papillomavirus Community in Healthy Persons, Defined by Metagenomics Analysis of Human Microbiome Project Shotgun Sequencing Data Sets. J. Virol..

[207] Sun X., Fu P., Xie L., Chai S., Xu Q., Zeng L., Wang X., Jiang N., Sang M. (2021). Resveratrol inhibits the progression of cervical cancer by suppressing the transcription and expression of HPV E6 and E7 genes. Int. J. Mol. Med..

[208] Houillé B., Papon N., Boudesocque L., Bourdeaud E., Besseau S., Courdavault V., Enguehard-Gueiffier C., Delanoue G., Guérin L., Bouchara J.-P. (2014). Antifungal activity of resveratrol derivatives against *Candida* species. J. Nat. Prod..

[209] Simonetti G., Palocci C., Valletta A., Kolesova O., Chronopoulou L., Donati L., Di Nitto A., Brasili E., Tomai P., Gentili A. (2019). Anti-*Candida* Biofilm Activity of Pterostilbene or Crude Extract from Non-Fermented Grape Pomace Entrapped in Biopolymeric Nanoparticles. Molecules.

[210] Simonetti G., Brasili E., Pasqua G. (2020). Antifungal Activity of Phenolic and Polyphenolic Compounds from Different Matrices of *Vitis vinifera* L. against Human Pathogens. Molecules.

[211] Navarro-Martínez M.D., García-Cánovas F., Rodríguez-López J.N. (2006). Tea polyphenol epigallocatechin-3-gallate inhibits ergosterol synthesis by disturbing folic acid metabolism in *Candida albicans*. J. Antimicrob. Chemother..

[212] Gomes C., Silva A.C., Marques A.C., Sousa Lobo J., Amaral M.H. (2020). Biotechnology Applied to Cosmetics and Aesthetic Medicines. Cosmetics.

[213] Kordi M., Salami R., Bolouri P., Delangiz N., Asgari Lajayer B., van Hullebusch E.D. (2022). White biotechnology and the production of bio-products. Syst. Microbiol. Biomanuf..

[214] Thorakkattu P., Khanashyam A.C., Shah K., Babu K.S., Mundanat A.S., Deliephan A., Deokar G.S., Santivarangkna C., Nirmal N.P. (2022). Postbiotics: Current Trends in Food and Pharmaceutical Industry. Foods.

[215] Pérez-Rivero C., López-Gómez J.P. (2023). Unlocking the Potential of Fermentation in Cosmetics: A Review. Fermentation.

[216] Salminen S., Collado M.C., Endo A., Hill C., Lebeer S., Quigley E.M.M., Sanders M.E., Shamir R., Swann J.R., Szajewska H. (2021). The International Scientific Association of Probiotics and Prebiotics (ISAPP) consensus statement on the definition and scope of postbiotics. Nat. Rev. Gastroenterol. Hepatol..

[217] Duarte M., Oliveira A.L., Oliveira C., Pintado M., Amaro A., Madureira A.R. (2022). Current postbiotics in the cosmetic market—An update and development opportunities. Appl. Microbiol. Biotechnol..

[218] Oliveira A.L.S., Seara M., Carvalho M.J., de Carvalho N.M., Costa E.M., Silva S., Duarte M., Pintado M., Oliveira C., Madureira A.R. (2023). Production of Sustainable Postbiotics from Sugarcane Straw for Potential Food Applications. Appl. Sci..

[219] Ciardiello T., Pinto D., Marotta L., Giuliani G., Rinaldi F. (2020). Effects of Fermented Oils on Alpha-Biodiversity and Relative Abundance of Cheek Resident Skin Microbiota. Cosmetics.

[220] Cortés-Martín A., Selma M.V., Tomás-Barberán F.A., González-Sarrías A., Espín J.C. (2020). Where to Look into the Puzzle of Polyphenols and Health? The Postbiotics and Gut Microbiota Associated with Human Metabotypes. Mol. Nutr. Food Res..

[221] Nataraj B.H., Ali S.A., Behare P.V., Yadav H. (2020). Postbiotics-parabiotics: The new horizons in microbial biotherapy and functional foods. Microb. Cell Fact..

[222] Yan F., Cao H., Cover T.L., Whitehead R., Washington M.K., Polk D.B. (2007). Soluble proteins produced by probiotic bacteria regulate intestinal epithelial cell survival and growth. Gastroenterology.

[223] Yan F., Liu L., Dempsey P.J., Tsai Y.-H., Raines E.W., Wilson C.L., Cao H., Cao Z., Liu L., Polk D.B. (2013). A *Lactobacillus rhamnosus* GG-derived soluble protein, p40, stimulates ligand release from intestinal epithelial cells to transactivate epidermal growth factor receptor. J. Biol. Chem..

[224] Sadgrove N.J. (2022). Honest nutraceuticals, cosmetics, therapies, and foods (NCTFs): Standardization and safety of natural products. Crit. Rev. Food Sci. Nutr..

[225] Antignac E., Nohynek G.J., Re T., Clouzeau J., Toutain H. (2011). Safety of botanical ingredients in personal care products/cosmetics. Food Chem. Toxicol..

[226] Goyal N., Jerold F. (2023). Biocosmetics: Technological advances and future outlook. Environ. Sci. Pollut. Res..

[227] Herman A., Herman A.P. (2023). Biological Activity of Fermented Plant Extracts for Potential Dermal Applications. Pharmaceutics.

